# Enhanced editing efficiency in Arabidopsis with a LbCas12a variant harboring D156R and E795L mutations

**DOI:** 10.1007/s42994-024-00144-w

**Published:** 2024-03-26

**Authors:** Cuiping Xin, Dexin Qiao, Junya Wang, Wei Sun, Zhenghong Cao, Yu Lu, Yuanyuan Jiang, Yiping Chai, Xue-Chen Wang, Qi-jun Chen

**Affiliations:** State Key Laboratory of Plant Environmental Resilience, College of Biological Sciences, China Agricultural University, Beijing, 100193 People’s Republic of China

**Keywords:** Genome editing, CRISPR/Cas, Cas12a, ttLbCas12a ultra, Arabidopsis

## Abstract

Cas12a (Cpf1), a Class 2 Type V CRISPR/Cas nuclease, has several unique attributes for genome editing and may provide a valuable alternative to Cas9. However, a low editing efficiency due to temperature sensitivity and insufficient cleavage activity of the Cas12a nuclease are major obstacles to its broad application. In this report, we generated two variants, ttAsCas12 Ultra and ttLbCas12a Ultra harboring three (E174R, M537R, and F870L) or two (D156R and E795L) mutations, respectively, by combining the mutations from the temperature-tolerant variants ttAsCas12a (E174R) and ttLbCas12a (D156R), and those from the highly active variants AsCas12a Ultra (M537R and F870L) and LbCas12a Ultra (E795L). We compared editing efficiencies of the five resulting Cas12a variants (LbCas12a, ttLbCas12a, ttLbCas12a Ultra, AsCas12a Ultra, and ttAsCas12 Ultra) at six target sites of four genes in Arabidopsis (*Arabidopsis thaliana*). The variant ttLbCas12a Ultra, harboring the D156R and E795L mutations, exhibited the highest editing efficiency of all variants tested in Arabidopsis and can be used to generate homozygous or biallelic mutants in a single generation in Arabidopsis plants grown at 22 °C. In addition, optimization of ttLbCas12a Ultra, by varying nuclear localization signal sequences and codon usage, further greatly improved editing efficiency. Collectively, our results indicate that ttLbCas12a Ultra is a valuable alternative to Cas9 for editing genes or promoters in Arabidopsis.

**Supplementary Information:**

The online version contains supplementary material available at 10.1007/s42994-024-00144-w.

## Introduction

Cas12a (Cpf1), an endonuclease from Class 2 Type V clustered regularly interspaced short palindromic repeat (CRISPR)/CRISPR-associated nuclease (Cas) systems, has several unique attributes as a genome-editing tool (Makarova et al. 2020; Zetsche et al. 2015). First, unlike Class 2 Type II CRISPR/Cas systems, Cas12a nucleases are complexed with a single CRISPR RNA (crRNA) that does not require a trans-activating CRISPR RNA (tracrRNA) (Zetsche et al. 2015). The crRNA of Cas12a (∼42 nt) is shorter than the single guide RNA (sgRNA) of Cas9 (∼100 nt), making it more amenable to chemical synthesis and easier to deliver with other CRISPR reagents into cells as ribonucleoprotein (RNP) complexes (Banakar et al. 2020; Su et al. 2023). Second, identified Cas12a nucleases, such as LbCas12a from *Lachnospiraceae bacterium* and AsCas12a from *Acidaminococcus* sp., typically recognize a T-rich protospacer adjacent motif (PAM) sequence at the 5' end of the protospacer (Zetsche et al. 2015). This attribute facilitates targeting AT-rich genomic regions that would be challenging to target with the most commonly used SpCas9 from *Streptococcus pyogenes*, which prefers G-rich PAMs (Zetsche et al. 2015). Third, Cas12a induces double-strand breaks (DSBs) with 5' overhangs that are distal from its PAM sequence (Zetsche et al. 2015). Since Cas12a-induced mutations are distal from the PAM, and mismatches distal from the PAM between the guide sequence of the crRNA and its target site in genomic DNA are more frequently tolerated by Cas9 or Cas12, Cas12a may potentially repeatedly induce DSBs even after the initial target site has been altered via imprecise DNA repair. Consequently, mutations induced by Cas12a are biased toward larger deletions compared to the mutations typically produced by Cas9 (Zetsche et al. 2015). In addition, repeated DNA cleavage facilitates releasing homology-directed repair (HDR) donors from genomic DNA and prolonging the time-window of HDR prior to repair via the non-homologous end joining (NHEJ) pathway. Finally, Cas12a proteins can process precursor crRNA (pre-crRNA) arrays, which can be harnessed for multiplex genome editing from a single transcript (Zetsche et al. 2017). Thus, Cas12a-induced genome editing could be a valuable alternative to Cas9.

Like Cas9, Cas12a has been employed for genome editing in plants (Bernabe-Orts et al. 2019; Lee et al. 2019; Li et al. 2018, 2020; Malzahn et al. 2019; Merker et al. 2020; Schindele et al. 2023; Schindele and Puchta 2020; Su et al. 2023; Tang et al. 2017; Wang et al. 2017, 2021; Wolter and Puchta 2019; Xu et al. 2017, 2019; Zhang et al. 2022; Zhong et al. 2018; Zhou et al. 2023). However, insufficient efficiency remains a major obstacle for its broad application (Guo et al. 2022; Lee et al. 2019; Liu et al. 2019a; Ma et al. 2022; Toth et al. 2020; Zhang et al. 2021). The optimal temperature for Cas12a activity is not compatible with plant cultivation, such as 22ºC for Arabidopsis (*Arabidopsis thaliana*), resulting in lower editing efficiency in plants compared to that in animals (Malzahn et al. 2019; Schindele and Puchta 2020). A heat-shock treatment has been used to overcome such temperature sensitivity in Arabidopsis (Blomme et al. 2022; Kurokawa et al. 2021; LeBlanc et al. 2018; Malzahn et al. 2019). However, the heat shock is typically applied to primary transformants (T_1_) or subsequent generations (T_2_) rather than T_0_ plants, thus limiting applications for efficient generation of homozygous or biallelic mutants in a single generation. Notably, utilizing Cas12a variants that can tolerate lower temperatures and retain high activity is an efficient strategy to overcome this problem in plants such as Arabidopsis (Merker et al. 2020; Schindele and Puchta 2020). Aside from temperature sensitivity, improving the cleavage activity of Cas12a is required and has been the focus of many efforts (Guo et al. 2022; Huang et al. 2022; Lee et al. 2019; Liu et al. 2019a; Ma et al. 2022; Toth et al. 2020; Zhang et al. 2021). The highly active AsCas12a variant harboring M537R and F870L mutations, named AsCas12a Ultra, was identified via directed evolution of AsCas12a in bacteria (Zhang et al. 2021). Likewise, the variant LbCas12a Ultra harboring the E795L mutation, an equivalent of AsCas12a Ultra, was reported (Zhang et al. 2023, 2021).

In this report, we generated two variants, ttAsCas12 Ultra and ttLbCas12a Ultra, harboring three (E174R, M537R, and F870L) or two (D156R and E795L) mutations, respectively, by combining the mutations reported for the low-temperature-tolerant variants ttAsCas12a (E174R) and ttLbCas12a (D156R) and the mutations from the highly active variants AsCas12a Ultra (M537R, and F870L) and LbCas12a Ultra (E795L). We compared the editing efficiency of the five resulting Cas12a variants at six target sites for four genes in Arabidopsis. Our results demonstrate that ttLbCas12a Ultra showed the highest editing efficiency in Arabidopsis and can be further optimized by varying nuclear localization signal sequences and codon usage; we anticipate that this new variant will add to the genome-editing toolbox available in Arabidopsis and other plants.

## Results

### The LbCas12a variant ttLbCas12a Ultra achieved the highest editing efficiency among five variants in Arabidopsis

**Fig. 1:**
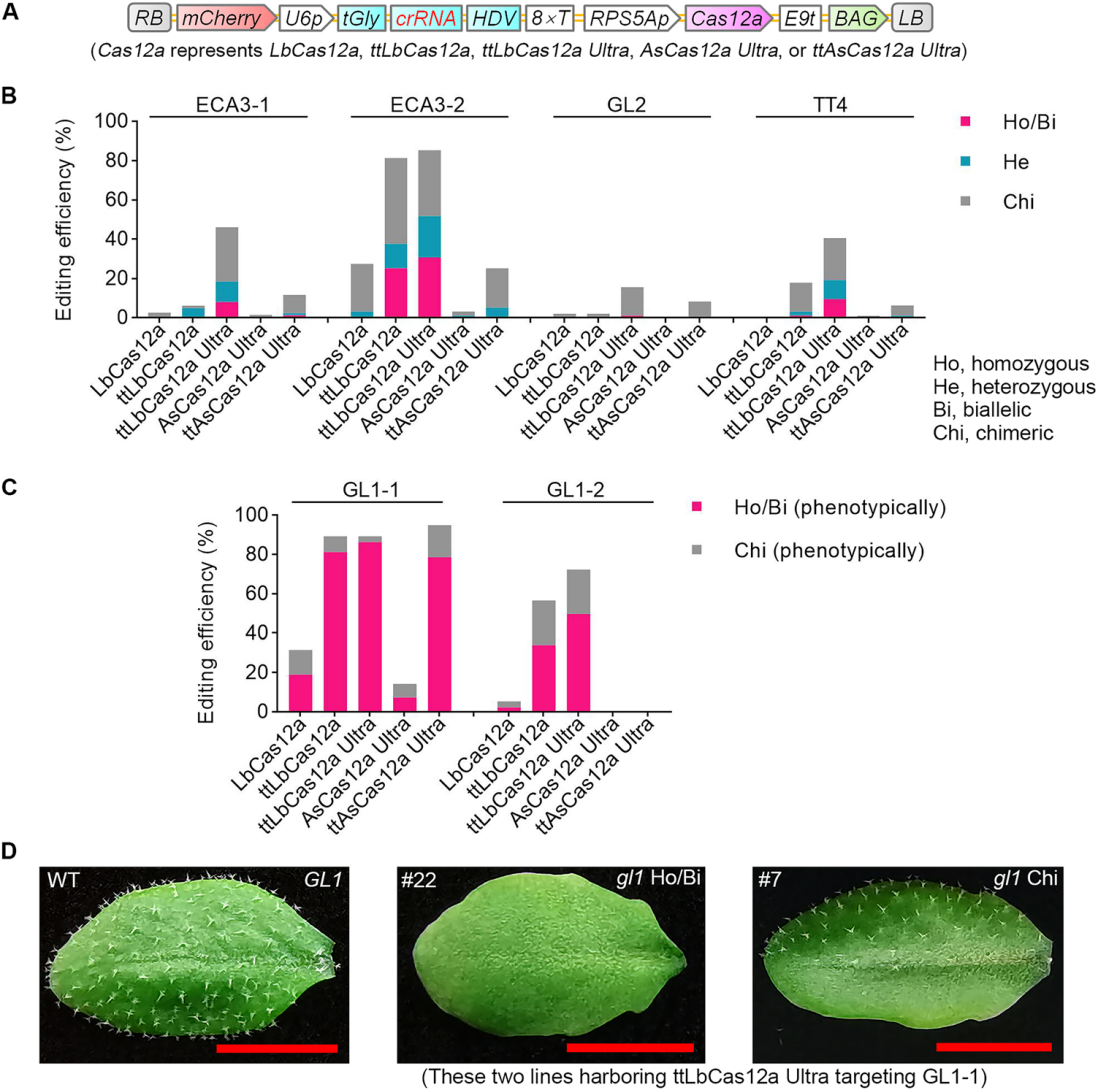
The LbCas12a variant ttLbCas12a Ultra, harboring the mutations D156R and E795L, confers higher editing efficiency than other variants at six targets in four genes of Arabidopsis. **A** T-DNA structures of binary vectors harboring five *Cas12a* genes or variants. RB and LB, T-DNA right and left border, respectively. U6p, Arabidopsis *U6-26* gene promoter; tGly, tRNA(Gly); HDV, hepatitis delta virus ribozyme; U6t, U6-26 terminator; RPS5Ap, Arabidopsis *RPS5A* promoter; rbcS-E9t, pea *rbcS E9* terminator; BAG, Bar-F2A-GAT, Basta and glyphosate-resistant marker gene used for selection. **B** Comparison of the editing efficiencies of five Cas12a variants at four targets in three genes. Mutations in each line were analyzed by deep sequencing of PCR amplicons. The editing efficiency represents the ratio of the number of plants with at least one edited allele to the total number of transgenic plants. Ho, homozygous mutants; Bi, biallelic mutants; He, heterozygous mutants; Chi, chimeric mutants. **C** Editing efficiencies of five Cas12a variants at two targets in *GL1*. Mutations in each line were determined based on completely or partially glabrous phenotypes. The editing efficiency represents the ratio of the number of plants with a glabrous phenotype resembling that of the indicated line to the total number of transgenic plants. **D** Representative glabrous phenotypes used to determine mutation types. Scale bar, 0.5 cm

To investigate whether Cas12a variants harboring mutations from low-temperature-tolerant or highly active variants have better editing efficiency than the currently used Cas12 endonucleases in Arabidopsis, we conducted parallel tests for five Cas12a variants: LbCas12a, ttLbCas12a (D156R), ttLbCas12a Ultra (D156R, E795L), AsCas12a Ultra (M537R, F870L), and ttAsCas12a Ultra (E174R, M537R, F870L). We tested the efficiency of each variant at six previously reported target sites for four genes: *GLABRA 1* (*GL1*), *GL2*, *TRANSPARENT TESTA 4* (*TT4*), and *ENDOPLASMIC RETICULUM-TYPE CALCIUM-TRANSPORTING ATPASE 3* (*ECA3*) (Malzahn et al. 2019; Schindele and Puchta 2020; Zhang et al. 2022). We used the *RIBOSOMAL PROTEIN 5A* (*RPS5A*) promoter to express each *Cas12a* variant, and the *U6 SMALL NUCLEOLAR RNA26* (*U6-26*) promoter to express each crRNA, which was flanked by a transfer RNA (tGly) and the hepatitis delta virus (HDV) ribozyme at their 5' and 3' ends, respectively, for precise processing (Fig. 1A) (Gao and Zhao 2014; Tang et al. 2017; Xie et al. 2015; Zhang et al. 2022). The construct also harbors an expression cassette for the red fluorescent protein gene *mCherry*, conferring a visible selection marker for the presence of the transgene (Dong et al. 2016).

We assessed the presence of homozygous, biallelic, or heterozygous mutations by high-throughput sequencing and calculated the editing efficiencies of each LbCas12a variant. We obtained the following percentages of plants with the desired mutation types among primary transformants with the LbCas12a variants LbCas12a, ttLbCas12a, and ttLbCas12a Ultra: 0% (0/77), 4.9% (4/81), and 18.4% (16/87), respectively, at the *ECA3-1* target; 3.2% (3/95), 37.5% (36/96), and 51.6% (49/95), respectively, at the *ECA3-2* target; 0 (0/96), 0 (0/96), and 1.0% (1/96), respectively, at the *GL2* target site; and 0 (0/95), 3.2% (3/95), and 19.1% (18/94), respectively, at the *TT4* target site (Fig. 1B; Table S1). We estimated homozygous or biallelic mutations at the *GL1-1* and *GL1-2* target sites based on the glabrous phenotype, yielding editing efficiencies for LbCas12a, ttLbCas12a, and ttLbCas12a Ultra of 18.8% (75/398), 81.2% (233/287), and 86.2% (326/378), respectively, at the *GL1-1* target site; and 2.2% (9/416), 33.8% (98/290), and 49.6% (138/278), respectively, at the *GL1-2* target site (Fig. 1C, D; Table S2). These results indicate that ttLbCas12a greatly enhances editing efficiency, compared to the original version, in Arabidopsis (Fig. 1B, C; Tables S1, S2), which is consistent with a previous report (Schindele and Puchta 2020). Importantly, our results indicate that ttLbCas12a Ultra further enhances the activity of ttLbCas12a and exhibits the highest editing efficiency at all six target sites tested (Fig. 1B, C; Tables S1, S2).

We also tested variants of AsCas12a: ttAsCas12a Ultra outperformed AsCas12a Ultra (Fig. 1B, C; Tables S1, S2). The editing efficiency of ttAsCas12a Ultra was, however, much lower than that of ttLbCas12a Ultra (Fig. 1B, C; Tables S1, S2), suggesting that AsCas12a activity is much more sensitive to temperature than that of LbCas12a (Schindele and Puchta 2020).

### Optimization of *crRNA* expression using two additional U6 cassettes

**Fig. 2:**
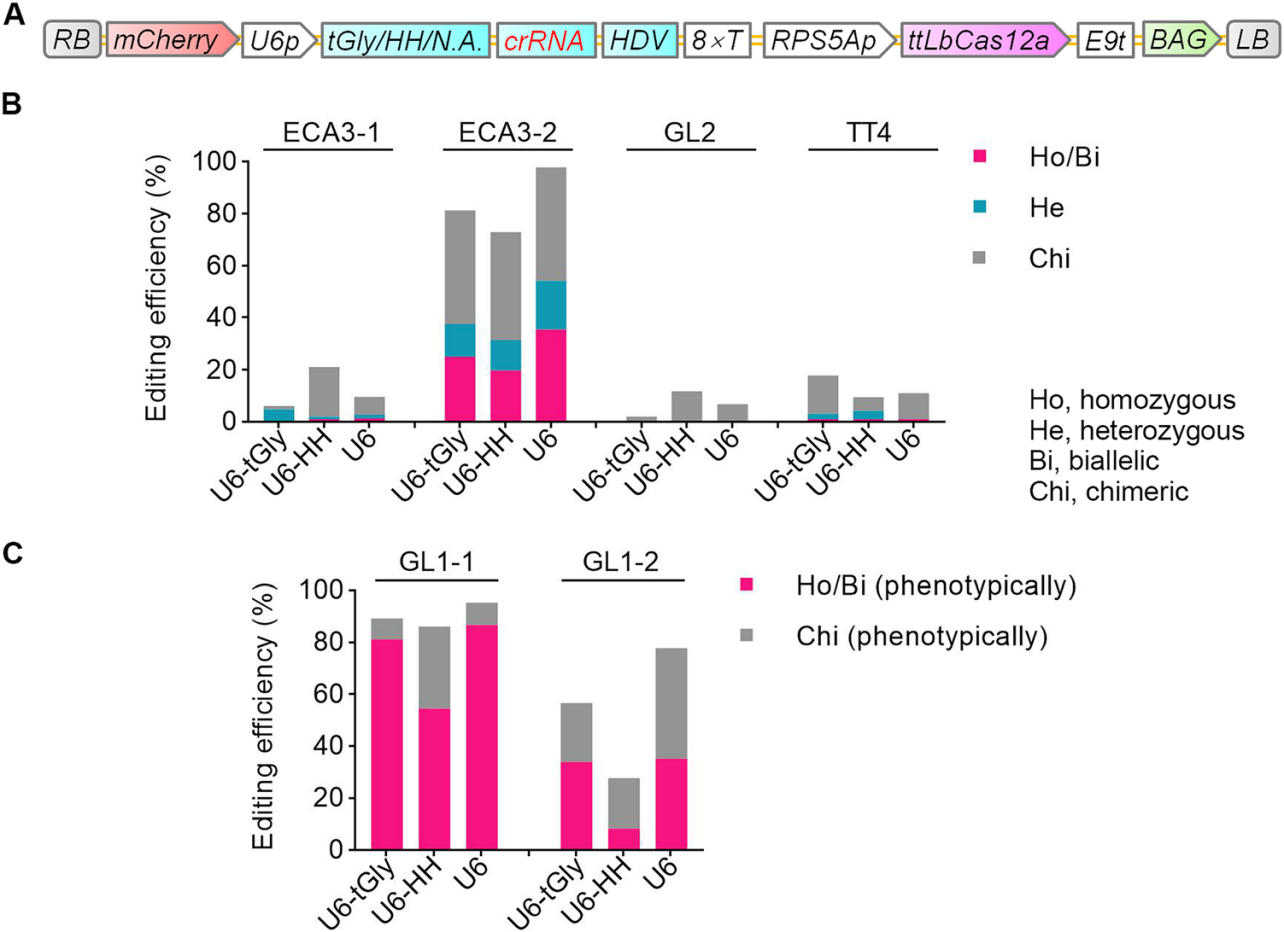
Comparison of the editing efficiencies of three different U6 cassettes for the expression of crRNA genes at six targets in four genes of Arabidopsis. **A** T-DNA structures of the three different crRNA expression cassettes. HH, hammerhead ribozyme. N.A., not available. See Fig. 1 for the other abbreviations. **B** Comparison of the editing efficiencies of three different U6 expression cassettes at four target sites in three genes. Mutations in each line were analyzed by deep sequencing of PCR amplicons. The editing efficiency represents the ratio of the number of plants with at least one edited allele to the total number of transgenic plants. Ho, homozygous mutants; Bi, biallelic mutants; He, heterozygous mutants; Chi, chimeric mutants. The data from *U6-tGly* are the same as in Fig. 1. **C** Comparison of the editing efficiencies of three different U6 expression cassettes at two targets in *GL1*. Mutations in each line were determined based on completely or partially glabrous phenotypes. The editing efficiency represents the ratio of the number of plants with a glabrous phenotype resembling that of the indicated line to the total number of transgenic plants

We asked whether the editing efficiency by ttLbCas12a can be improved by optimizing the expression of the *crRNA*. We thus tested two additional *crRNA* expression cassettes based on variations in the *U6-26* promoter, resulting in three cassettes (the original *U6-tRNA*, *U6-HH*, and *U6,* where HH represents hammerhead ribozyme) and using the ttLbCas12a variant (Fig. 2A). We scored the presence of mutations (homozygous, biallelic, or heterozygous) by high-throughput sequencing, resulting in percentages of edited plants with the *U6-tRNA*, *U6-HH*, or *U6* cassette of 4.9% (4/81), 2.1% (2/95), and 2.8% (2/72), respectively, at the *ECA3-1* target site; 37.5% (36/96), 31.3% (30/96), and 54.2% (52/96), respectively, at the *ECA3-2* target site; 0 (0/96), 0 (0/93), and 0 (0/87), respectively, at the *GL2* target site; and 3.2% (3/95), 4.3% (4/94), and 1.1% (1/90), respectively, at the *TT4* target site (Fig. 2B; Table S3).

We also determined the percentage of homozygous or biallelic mutations based on the glabrous phenotype, revealing percentages of edited plants with the *U6-tRNA*, *U6-HH*, or *U6* cassette of: 81.2% (233/287), 54.5% (145/266), and 86.6% (291/336), respectively, at the *GL1-1* target site; and 33.8% (98/290), 8.3% (15/181), and 35.1% (52/148), respectively, at the *GL1-2* target site (Fig. 2C; Table S4).

Based on the number of homozygous or biallelic mutations, we concluded that the *U6* cassette achieved higher (4 out of 6 targets) or similar (2 out of 6 targets) editing efficiency compared to *U6-tRNA* or *U6-HH* versions (Fig. 2; Table S3, S4). Since homozygous or biallelic mutations are always the desired mutation types, these results demonstrate that the *U6* cassette outperformed the other two tested expression cassettes.

### Optimization of *crRNA* expression using RNA polymerase II promoters

**Fig. 3:**
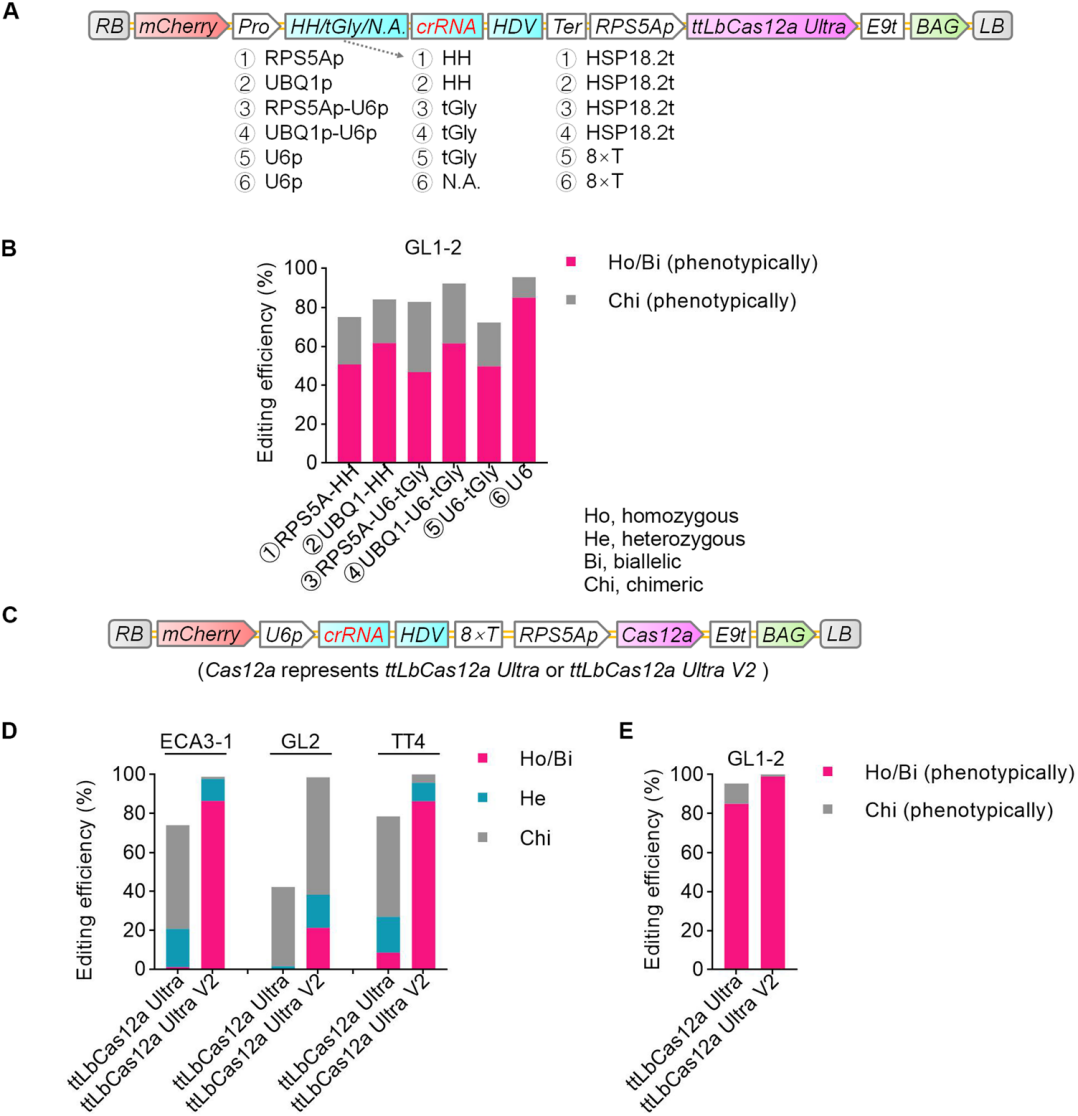
Comparison of the editing efficiencies of RNA Pol II, III, and composite promoters for the expression of crRNA. **A** T-DNA structures of six different expression cassettes. See Figs. 1 or 2 for abbreviations. **B** Editing efficiencies of six different crRNA cassettes at the *GL1-2* target. Mutations in each line were determined based on completely or partially glabrous phenotypes. The editing efficiency represents the ratio of the number of plants with a glabrous phenotype resembling that of the indicated line to the total number of transgenic plants. The data from *U6-tGly* are the same as in Fig. 1. **C** T-DNA structures of the two versions of *ttLbCas12a Ultra*. See Fig. 1 for abbreviations. **D**, **E** Editing efficiencies of the two versions of *ttLbCas12a Ultra*

To investigate whether the editing efficiency by ttLbCas12a Ultra can be further improved by optimizing the expression of *crRNA*s, we tested six additional *crRNA* expression cassettes based on RNA polymerase II (Pol II) promoters: *RPS5A*, *UBIQUITIN 1* (*UBQ1*), a combination of *RPS5A* and *U6*, and a combination of *UBQ1* with *U6* (Fig. 3A). For phenotypically homozygous or biallelic mutations, we ordered the six *crRNA* expression cassettes based on decreasing percentage of edited plants as follows: *U6* > *UBQ1-HH* > *UBQ1-U6-tGly* ≥ *RPS5A-HH* > *U6-tGly* > *RPS5A-U6-tGly* (Fig. 3B; Table S5). The results shown in Fig. 3B demonstrate that the four cassettes harboring two Pol II promoters worked well to express *crRNA*s, although with lower efficiency than the *U6* cassette.

### Optimization of ttLbCas12a Ultra by varying NLS sequences and codon usage

Since optimized nuclear localization signal (NLS) sequences enhanced prime editing efficiency (Chen et al. 2021), and the *ttLbCas12a Ultra* sequence was codon-optimized with monocot plants, we generated another version of *ttLbCas12a Ultra* that harbored the same NLS sequences as in PEmax (Chen et al. 2021) and was codon-optimized for dicot species, which we named *ttLbCas12a Ultra V2* (Fig. 3C; Table S6). The optimized NLS sequences were composed of a bipartite SV40 NLS at both terminals and an additional c-Myc NLS at C-terminal. Indeed, we obtained much more edited plants when expressing *ttLbCas12a Ultra V2* than with *ttLbCas12a Ultra*, indicating that editing efficiency can be further improved in Arabidopsis by optimizing NLS sequences and the codon usage of the nuclease gene (Fig. 3D; Table S6).

### Mutations in T_1_ plants are heritable to the subsequent generation

The *mCherry* cassette in the Cas12a vectors provided an effective strategy for reliably isolating *Cas12*-free Arabidopsis plants (Gao et al. 2016). Indeed, seeds harboring the transgene with the *mCherry* cassette display strong red fluorescence, while the absence of red fluorescence indicates segregation of the T-DNA, resulting in T-DNA-free seeds (Gao et al. 2016). Before analyzing the heritability of each mutation, we first isolated T-DNA-free seeds, based on the lack of red fluorescence.

To test whether mutations at the *ECA3-1* and *ECA3-2* target sites are heritable, we isolated T-DNA-free T_2_ seeds from six and nine T_1_ biallelic mutant plants, respectively. Since Cas12a-induced mutations at the *ECA3-1* and *ECA3-2* target sites disrupted the *Bgl*II and *Eco*RV restriction enzyme sites, respectively, we employed restriction fragment length polymorphism (RFLP) analysis to identify mutations. RFLP analysis indicated that all T-DNA-free T_2_ plants harbor homozygous or biallelic mutations, as evidenced by the lack of DNA cleavage (Fig. S1, S2; Table S7). These results demonstrate that mutations in *ECA3* present in the T_1_ plants are heritable.

**Fig. 4:**
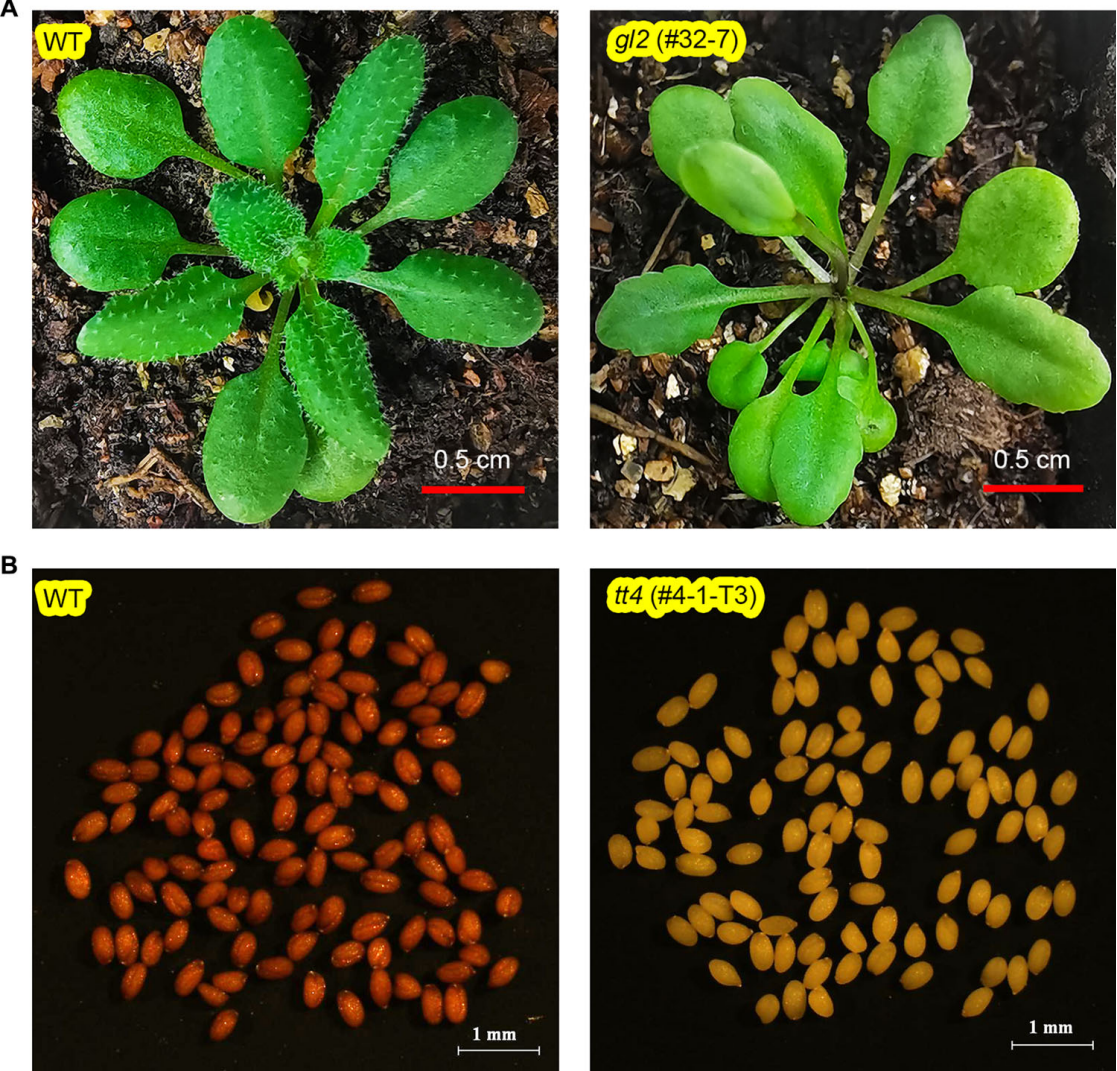
Phenotypes of T-DNA-free homozygous *gl2* or *tt4* mutants derived from T1 plants harboring *ttLbCas12a Ultra* and the U6-tRNA cassette. **A** Glabrous phenotype of a T-DNA-free T2 homozygous *gl2* mutant (right) compared to the wild type (WT; left). **B** Yellow seed coat phenotype of a T-DNA-free T3 homozygous *tt4* mutant (right) compared to the WT (left)

To provide evidence that mutations at the *GL2* target are heritable, we isolated 30 T-DNA-free T_2_ seeds from the only identified T_1_ biallelic mutant plant, which surprisingly did not exhibit a glabrous phenotype (Table S7). High-throughput sequencing analysis of the resulting 30 T_2_ plants showed that they carry homozygous or biallelic mutations identical to the two mutations detected in the T_1_ plant (Table S8). We observed that six plants homozygous for an 8-bp deletion at the target site exhibit the glabrous phenotype (Fig. 4A), whereas the remaining six plants homozygous for a 21-bp insertion and 18 plants with biallelic mutations did not show a glabrous phenotype (Table S8). These results indicate that the 21-bp insertion, which leads to an insertion of seven amino acids, does not affect GL2 function, explaining the lack of a glabrous phenotype of the original T_1_ plant. These results do, however, demonstrate that the introduced mutations in *GL2* are heritable.

We performed a similar analysis of mutations at the *TT4* target site; to this end, we isolated T-DNA-free T_2_ seeds from seven T_1_ homozygous or biallelic mutant plants. All T_2_ seeds were yellow rather than dark brown (Fig. 4B and Table S8), a phenotype typical of *tt4* mutants (Malzahn et al. 2019). We conclude that all T_2_ progeny are homozygous or biallelic *tt4* mutants, demonstrating that mutations present in T_1_ plants at the *TT4* target site are heritable.

We also isolated T-DNA-free T_2_ seeds from ten (*GL1-1*) and five (*GL1-2*) T_1_ phenotypically homozygous or biallelic mutant plants in which *GL1* was targeted for editing (Table S10). We determined that all T_2_ plants are completely glabrous (Table S10). Sanger sequencing of the target site in individual T_2_ plants confirmed that they harbor homozygous or biallelic mutations in *GL1* (Tables S11, S12). These results demonstrate that mutations present in T_1_ plants at the *GL1* locus are heritable.

### Off-target mutations were not detected in mutant plants

High editing efficiency usually means high off-target mutagenesis. We selected the most efficient two targets, *GL1-1* and *GL1-2*, to analyze off-target mutations. We searched potential off-target sites of the *GL1-1* target and obtained two harboring 3 or 4 mismatches in *At5G40330* or *At1G22640*, respectively (Table S13). We also obtained an off-target site with 5 mismatches of the *GL1-2* target (Table S13). We amplified PCR fragments spanning off-target sites from 36 *gl1-1* and 36 *gl1-2* T1 mutant plants that harbor homozygous or biallelic mutations and analyzed mutations by Sanger sequencing. We detected no off-target mutations (Table S13), indicating that careful selection of targets will be able to avoid off-target mutagenesis induced by highly efficient LbCas12a variants.

## Discussion

Temperature sensitivity and insufficient cleavage activity of Cas12a nucleases limit editing efficiency of targets in plants and many efforts have been made to improve these restrictions (Guo et al. 2022; Huang et al. 2022; Lee et al. 2019; Liu et al. 2019a; Ma et al. 2022; Zhang et al. 2023, 2021). Two LbCas12a Ultra variants have previously been reported: one harbors a single E795L mutation (Zhang et al. 2021) and another harbors the two mutations N527R and E795L (Huang et al. 2022). In this report, we combined the D156R mutation from the low-temperature-tolerant variant and the E795L mutation to greatly enhance editing efficiency of LbCas12a (Fig. 1). Our results are consistent with a previous report that introducing mutations from a low-temperature-tolerant variant into a LbCas12a Ultra variant greatly enhanced editing efficiency of LbCas12a (Huang et al. 2022). The main difference with the previous report is that we used the variant harboring the single E795L mutation, whereas Huang et al. used the variant harboring the two mutations N527R and E795L. However, although E795L enhanced the editing efficiency, the mutation N527R showed the detrimental effect on LbCas12a activity, and the variant harboring the two mutations failed to show improved editing efficiency in human cells (Zhang et al. 2023). In the future, it will be interesting to compare the ttLbCas12a Ultra variant generated in this report to other highly active variants, such as Cas12a-Plus (Huang et al. 2022), hyper-Cas12a (Guo et al. 2022), iCas12a (Ma et al. 2022), and LbCas12a-RRV (Zhang et al. 2023) for use in plants. These comparisons will help generate more Cas12a variants with higher editing efficiency.

Since processing of *tRNA-cRNA*s or *HH-crRNA*s is not complete (Gao and Zhao 2014; Xie et al. 2015), it was not surprising that the *U6* expression cassette outperformed the other two *U6:crRNA* expression cassettes (Fig. 2). These results suggest that residual *tRNA-crRNA* or *HH-crRNA* fusions resulting from incomplete processing of the *tRNA* or ribozyme have low editing activity compared to fully processed crRNAs with clean 5' ends. Our results indicate that cassettes based on two Pol II promoters to express *crRNA*s worked well, although with lower efficiency than the *U6-*based cassette (Fig. 3). Since Pol II promoters can drive transcription of much longer genes than Pol III promoters, such as *U6*, we suspect that the two Pol II promoters will be useful for multiplex genome editing based on expressing a single transcript consisting of multiple crRNAs in tandem (Zetsche et al. 2017). Excitingly, optimization of ttLbCas12a Ultra by varying nuclear localization signal sequences and codon usage can further greatly enhance editing efficiency. In the future, it will be interesting to test which of the two factors is decisive for the enhancement.

We also demonstrated that mutations at all six target sites for four genes were transmitted from T_1_ plants to their T-DNA-free T_2_ progeny (Fig. 4, S1, S2; Tables S7–S12). Our results indicate that optimized LbCas12a tools efficiently generate homozygous or biallelic mutants in a single generation following the transformation of Arabidopsis plants grown at 22 °C. Since mutations induced by Cas12a are biased toward larger deletions than those typically produced by Cas9 (Tables S8, S11, S12), ttLbCas12a Ultra may provide a valuable alternative to Cas9 for editing promoters in Arabidopsis and other plants.

In conclusion, a LbCas12a variant, ttLbCas12a Ultra, harboring the D156R and E795L mutations from a low-temperature-tolerant variant and a highly active variant, respectively, achieved high editing efficiency in Arabidopsis grown at 22 °C; ttLbCas12a Ultra can be further optimized by varying nuclear localization signal sequences and codon usage; ttLbCas12a Ultra may thus be used to efficiently generate homozygous or biallelic mutants in a single generation in Arabidopsis grown at 22 °C, providing a valuable alternative to Cas9 for editing genes or promoters in Arabidopsis.

## Materials and methods

### Vector construction

All primers used in this study are listed in Table S14, and the sequences of all target sites are listed in Table S15. Vectors described in this study, together with their annotated sequences, are available from Addgene.

The *Hin*dIII-*Sac*I fragment including LbCas12a of pHRLbA (Zhang et al. 2022) was used to replace the *Hin*dIII-*Sac*I fragment of pC2BG, resulting in pBG-RLbA0. A short insert was generated by annealing the two primers oHA-BS-F and oHA-BS-R to replace the *Bam*HI-*Sac*I fragment of pBG-RLbA0, resulting in pBG-Lb01. The D156R mutation was introduced into LbCas12a, resulting in ttLb12a. The E795L mutation was similarly introduced into ttLbCas12a, resulting in ttLb12a Ultra. The E174R mutation was introduced into a synthetic fragment of AsCas12a Ultra, resulting in ttAsCas12a Ultra. The *Xba*I-*Sac*I fragment of pBG-Lb01 was replaced with a *Xba*I-*Sac*I fragment encoding ttLbCas12a, ttLbCas12a Ultra, AsCas12a Ultra, and ttAsCas12a Ultra, resulting in pBG-ttLb01, pBG-ttLbU01, pBG-AsU01P1, and ttAsU01P1, respectively. The *Avr*II-*Bsa*I and *Bsa*I-*Bsa*I fragments of pBG-AsU01P1 and pBG-ttAsU01P1 were replaced with a synthetic fragment digested with *Avr*II and *Aar*I, resulting in pBG-AsU01 and pBG-ttAsU01, respectively. Two synthetic fragments digested with *Nco*I and *Spe*I were used to replace the *Nco*I-*Spe*I fragment of pBG-ttLb01, resulting in pBG-ttLb02 and pBG-ttLb, respectively.

The crRNA sequences were obtained by annealing two primers for each target site: oECA3.1-F/R, oECA3.2-F/R, oGL2-F/R, oTT4-F/R, oGL1.1-F/R, and oGL1.2-F/R. The resulting double-stranded DNA sequence was used to replace the *Bsa*I-*Bsa*I fragments of pBG-Lb01, pBG-ttLb01, pBG-ttLb02, pBG-ttLb, pBG-ttLbU01, pBG-AsU01, and pBG-ttAsU01, resulting in 42 final vectors used for Agrobacterium (*Agrobacterium tumefaciens*)-mediated Arabidopsis transformation (Table S16).

A short insert generated by annealing the two primers oiCBAHS-F and oiCBAHS-R was used to replace the I-*Ceu*I-*Spe*I fragment of pBG-ttLbU01, resulting in pBG-ttLb12U-dA. The *Bsa*I-*Avr*II fragment of pBG-ttLb12U-dA was replaced with the *Hin*d3-*Avr*II fragment of pBG-ttLbU01, resulting in pBG-ttLb12U-mCh. The *Hin*d3-*Spe*I fragment of pBG-ttLb12U-mCh was replaced with the *Hin*d3-*Spe*I fragments of U6, RPS5A-HH, UBQ1-HH, RPS5A-tR, and UBQ1-tR, resulting in pBG-ttLbU, pBG-ttLbU03, pBG-ttLbU04, pBG-ttLbU05, and pBG-ttLbU06, respectively. The *Xba*I-*Sac*I fragment of pBG-ttLbU was replaced with a synthetic ttLbCas12a Ultra V2 digested with *Xba*I and *Sac*I, resulting in pBG-ttLbUV2. A small insert was prepared by annealing the two primers oGL1.2-F and oGL1.2-R to replace the *Bsa*I-*Bsa*I fragments of pBG-ttLbU03, pBG-ttLbU04, pBG-ttLbU05, pBG-ttLbU06, pBG-ttLbU, and pBG-ttLbUV2, resulting in six final vectors used for Agrobacterium-mediated Arabidopsis transformation (Table S16). Three pairs of final vectors derived from pBG-ttLbU or pBG-ttLbUV2 and harboring *ECA3-1*, *GL2*, or *TT4* targets were also generated with the crRNA sequences prepared by annealing two primers for each target site: oECA3.1-F/R, oGL2-F/R, and oTT4-F/R.

### Generation of transgenic plants and analysis of mutations

Each of the 54 vectors (Table S16) were individually introduced into Agrobacterium strain GV3101. Transgenic plants were generated in the *Arabidopsis thaliana* accession Col-0 via the floral dip method. Primary (T_1_) transformants were selected on Murashige and Skoog (MS) plates containing 100 μM glyphosate before transferring the resistant seedlings to soil.

Genomic DNA was extracted from T_1_ transgenic plants grown in soil. To analyze mutations at the *ECA3-1*, *ECA3-2*, *GL2*, and *TT4* targets by high-throughput sequencing, fragments surrounding the above target sites were amplified by PCR with the primer pairs ECA3-1-NGSF/R, ECA3-2-NGSF/R, GL2-NGSF/R, and TT4-NGSF/R, respectively. The Hi-Tom platform was used to analyze sequencing results (Liu et al. 2019b). The mutation efficiency was calculated based on the ratio between the number of plants with mutations to the total number of transgenic plants. When ≥ 95% high-throughput sequencing reads from a line presented the same type of mutation, this line was scored as a homozygous (Ho) mutant; When ≥ 95% high-throughput sequencing reads from a given line presented more than one type of mutation, this line was considered biallelic (Bi). For non-homozygous and non-biallelic mutants, when ≥ 45% or < 45% high-throughput sequencing reads from a line presented the same type of mutation, these lines were scored as heterozygous (He) or chimeric (Chi) mutants, respectively. For the target sites *GL1-1* and *GL1-2*, the editing efficiency was analyzed by counting the number of glabrous plants.

For analysis of off-target mutations in *At5G40330*, *At1G22640*, and *At5G14750*, primer pairs At5G40330-F/R, At1G22640-F/R, and At5G14750-F/R, respectively, were used to amplify PCR fragments spanning the potential off-target sites. The PCR fragments were analyzed for possible mutations by Sanger sequencing.

### Analysis of heritable mutations

To analyze heritable mutations, T_2_ seeds lacking red fluorescence were selected, and then the presence of mutations in these T-DNA-free T_2_ plants was analyzed. To analyze heritable mutations in *ECA3*, fragments surrounding the target sites *ECA3-1* and *ECA3-2* were amplified by PCR with the primer pairs ECA3-1-IDF/IDR and ECA3-2-IDF/IDR, respectively. The PCR products from the *ECA3-1* and *ECA3-2* target sites were analyzed by restriction fragment length polymorphism (RFLP) analysis through direct digestion with *Bgl*II or *Eco*RV, respectively. For analysis of heritable mutations in *GL2* by high-throughput sequencing, fragments surrounding the target site at *GL2* were amplified by PCR with the primer pair GL2-NGSF/R. The Hi-Tom platform was used to analyze the sequencing results (Liu et al. 2019b). For analysis of heritable mutations in *TT4*, seed coat color was scored. For analysis of heritable mutations in *GL1*, the glabrous phenotype of plants was observed, and then fragments surrounding the target sites of *GL1-1* and *GL1-2* were amplified with the primer pairs GL1-1-IDF/IDR and GL1-2-IDF/IDR, respectively, and the purified PCR products were submitted for Sanger sequencing with primers GL1-1-IDF and GL1-2-IDF, respectively. DSDecode was used to analyze the results of Sanger sequencing (Liu et al. 2015).

### Supplementary Information

Below is the link to the electronic supplementary material.

Supplementary file1 (PDF 816 KB)

## Data Availability

Annotated sequences of vectors described in this study and deep sequencing data are available from the corresponding author on reasonable request.

## References

[CR1] Banakar R, Schubert M, Collingwood M, Vakulskas C, Eggenberger AL, Wang K (2020). Comparison of CRISPR-Cas9/Cas12a ribonucleoprotein complexes for genome editing efficiency in the rice phytoene desaturase (OsPDS) gene. Rice.

[CR2] Bernabe-Orts JM, Casas-Rodrigo I, Minguet EG, Landolfi V, Garcia-Carpintero V, Gianoglio S (2019). Assessment of Cas12a-mediated gene editing efficiency in plants. Plant Biotechnol J.

[CR3] Blomme J, Develtere W, Kose A, Arraiza Ribera J, Brugmans C, Jaraba-Wallace J (2022). The heat is on: a simple method to increase genome editing efficiency in plants. BMC Plant Biol.

[CR4] Chen PJ, Hussmann JA, Yan J, Knipping F, Ravisankar P, Chen PF (2021). Enhanced prime editing systems by manipulating cellular determinants of editing outcomes. Cell.

[CR5] Dong D, Ren K, Qiu X, Zheng J, Guo M, Guan X (2016). The crystal structure of Cpf1 in complex with CRISPR RNA. Nature.

[CR6] Gao X, Chen J, Dai X, Zhang D, Zhao Y (2016). An effective strategy for reliably isolating heritable and Cas9-free arabidopsis mutants generated by CRISPR/Cas9-mediated genome editing. Plant Physiol.

[CR7] Gao Y, Zhao Y (2014). Self-processing of ribozyme-flanked RNAs into guide RNAs in vitro and in vivo for CRISPR-mediated genome editing. J Integr Plant Biol.

[CR8] Guo LY, Bian J, Davis AE, Liu P, Kempton HR, Zhang X (2022). Multiplexed genome regulation in vivo with hyper-efficient Cas12a. Nat Cell Biol.

[CR9] Huang H, Huang G, Tan Z, Hu Y, Shan L, Zhou J (2022). Engineered Cas12a-Plus nuclease enables gene editing with enhanced activity and specificity. Bmc Biol.

[CR10] Kurokawa S, Rahman H, Yamanaka N, Ishizaki C, Islam S, Aiso T (2021). A simple heat treatment increases SpCas9-mediated mutation efficiency in Arabidopsis. Plant Cell Physiol.

[CR11] LeBlanc C, Zhang F, Mendez J, Lozano Y, Chatpar K, Irish VF, Jacob Y (2018). Increased efficiency of targeted mutagenesis by CRISPR/Cas9 in plants using heat stress. Plant J.

[CR12] Lee K, Zhang Y, Kleinstiver BP, Guo JA, Aryee MJ, Miller J (2019). Activities and specificities of CRISPR/Cas9 and Cas12a nucleases for targeted mutagenesis in maize. Plant Biotechnol J.

[CR13] Li S, Zhang X, Wang W, Guo X, Wu Z, Du W (2018). Expanding the scope of CRISPR/Cpf1-mediated genome editing in rice. Mol Plant.

[CR14] Li S, Zhang Y, Xia L, Qi Y (2020). CRISPR-Cas12a enables efficient biallelic gene targeting in rice. Plant Biotechnol J.

[CR15] Liu P, Luk K, Shin M, Idrizi F, Kwok S, Roscoe B (2019). Enhanced Cas12a editing in mammalian cells and zebrafish. Nucleic Acids Res.

[CR16] Liu Q, Wang C, Jiao X, Zhang H, Song L, Li Y (2019). Hi-TOM: a platform for high-throughput tracking of mutations induced by CRISPR/Cas systems. Sci China Life Sci.

[CR17] Liu W, Xie X, Ma X, Li J, Chen J, Liu YG (2015). DSDecode: a web-based tool for decoding of sequencing chromatograms for genotyping of targeted mutations. Mol Plant.

[CR18] Ma E, Chen K, Shi H, Stahl EC, Adler B, Trinidad M (2022). Improved genome editing by an engineered CRISPR-Cas12a. Nucleic Acids Res.

[CR19] Makarova KS, Wolf YI, Iranzo J, Shmakov SA, Alkhnbashi OS, Brouns SJJ (2020). Evolutionary classification of CRISPR-Cas systems: a burst of class 2 and derived variants. Nat Rev Microbiol.

[CR20] Malzahn AA, Tang X, Lee K, Ren Q, Sretenovic S, Zhang Y (2019). Application of CRISPR-Cas12a temperature sensitivity for improved genome editing in rice, maize, and Arabidopsis. BMC Biol.

[CR21] Merker L, Schindele P, Huang TK, Wolter F, Puchta H (2020). Enhancing in planta gene targeting efficiencies in Arabidopsis using temperature-tolerant CRISPR/LbCas12a. Plant Biotechnol J.

[CR22] Schindele P, Merker L, Schreiber T, Prange A, Tissier A, Puchta H (2023). Enhancing gene editing and gene targeting efficiencies in Arabidopsis thaliana by using an intron-containing version of ttLbCas12a. Plant Biotechnol J.

[CR23] Schindele P, Puchta H (2020). Engineering CRISPR/LbCas12a for highly efficient, temperature-tolerant plant gene editing. Plant Biotechnol J.

[CR24] Su H, Wang Y, Xu J, Omar AA, Grosser JW, Calovic M (2023). Generation of the transgene-free canker-resistant *Citrus sinensis* using Cas12a/crRNA ribonucleoprotein in the T0 generation. Nat Commun.

[CR25] Tang X, Lowder LG, Zhang T, Malzahn AA, Zheng X, Voytas DF (2017). A CRISPR-Cpf1 system for efficient genome editing and transcriptional repression in plants. Nat Plants.

[CR26] Toth E, Varga E, Kulcsar PI, Kocsis-Jutka V, Krausz SL, Nyeste A (2020). Improved LbCas12a variants with altered PAM specificities further broaden the genome targeting range of Cas12a nucleases. Nucleic Acids Res.

[CR27] Wang M, Mao Y, Lu Y, Tao X, Zhu JK (2017). Multiplex gene editing in rice using the CRISPR-Cpf1 system. Mol Plant.

[CR28] Wang W, Tian B, Pan Q, Chen Y, He F, Bai G (2021). Expanding the range of editable targets in the wheat genome using the variants of the Cas12a and Cas9 nucleases. Plant Biotechnol J.

[CR29] Wolter F, Puchta H (2019). In planta gene targeting can be enhanced by the use of CRISPR/Cas12a. Plant J.

[CR30] Xie K, Minkenberg B, Yang Y (2015). Boosting CRISPR/Cas9 multiplex editing capability with the endogenous tRNA-processing system. Proc Natl Acad Sci USA.

[CR31] Xu R, Qin R, Li H, Li D, Li L, Wei P, Yang J (2017). Generation of targeted mutant rice using a CRISPR-Cpf1 system. Plant Biotechnol J.

[CR32] Xu R, Qin R, Li H, Li J, Yang J, Wei P (2019). Enhanced genome editing in rice using single transcript unit CRISPR-LbCpf1 systems. Plant Biotechnol J.

[CR33] Zetsche B, Gootenberg JS, Abudayyeh OO, Slaymaker IM, Makarova KS, Essletzbichler P (2015). Cpf1 is a single RNA-guided endonuclease of a class 2 CRISPR-Cas system. Cell.

[CR34] Zetsche B, Heidenreich M, Mohanraju P, Fedorova I, Kneppers J, DeGennaro EM (2017). Multiplex gene editing by CRISPR-Cpf1 using a single crRNA array. Nat Biotechnol.

[CR35] Zhang L, Li G, Zhang Y, Cheng Y, Roberts N, Glenn SE (2023). Boosting genome editing efficiency in human cells and plants with novel LbCas12a variants. Genome Biol.

[CR36] Zhang L, Zuris JA, Viswanathan R, Edelstein JN, Turk R, Thommandru B (2021). AsCas12a ultra nuclease facilitates the rapid generation of therapeutic cell medicines. Nat Commun.

[CR37] Zhang Q, Zhang Y, Chai Y (2022). Optimization of CRISPR/LbCas12a-mediated gene editing in Arabidopsis. PLoS ONE.

[CR38] Zhong Z, Zhang Y, You Q, Tang X, Ren Q, Liu S (2018). Plant genome editing using FnCpf1 and LbCpf1 nucleases at redefined and altered PAM sites. Mol Plant.

[CR39] Zhou J, Liu G, Zhao Y, Zhang R, Tang X, Li L (2023). An efficient CRISPR-Cas12a promoter editing system for crop improvement. Nat Plants.

