# Supplementary material for: Enhanced editing efficiency in Arabidopsis with a LbCas12a variant harboring D156R and E795L mutations
Source: aBIOTECH. 2024 Mar 26;5(2):117–26. doi: 10.1007/s42994-024-00144-w (PMC11229449; doi:10.1007/s42994-024-00144-w)
Supplement: Supplementary file 1 — Supplementary file1 (PDF 816 KB) [file 42994_2024_144_MOESM1_ESM.pdf]

Enhanced editing efficiency in Arabidopsis with a LbCas12a variant harboring D156R and E795L mutations

Cuiping **Xin**[1], Dexin **Qiao**[1], Junya **Wang**[1], Wei **Sun**[1], Zhenghong **Cao**[1], Yu **Lu**[1], Yuanyuan **Jiang**[1], Yiping **Chai**[1], Xue-Chen **Wang**[1], Qijun **Chen**[1]

[1]State Key Laboratory of Plant Environmental Resilience, College of Biological Sciences, China Agricultural University, Beijing 100193, China

[*] Correspondence (email: qjchen@cau.edu.cn)

Supplementary Information

Table of contents

Fig. S1. RFLP analysis of mutations at the *ECA3-1* target site 3
Fig. S2. RFLP analysis of mutations at the *ECA3-2* target site 4
Table S1. Mutation efficiency of the five Cas12a variants at four target sites for three genes 5
Table S2. Mutation efficiency of the five Cas12a variants at the *GL1-1* and *GL1-2* target sites 6
Table S3. Mutation efficiency of three U6 cassettes 7
Table S4. Mutation efficiency of the three U6 cassettes at the *GL1-1* and *GL1-2* target sites 8
Table S5. Mutation efficiency of different promoters driving the expression of the crRNA 9
Table S6. Mutation efficiency of the two versions of *ttLbCas12a Ultra* 10
Table S7. Heritable mutations at the *ECA3-1*, *ECA3-2*, and *GL2* target sites in T-DNA-free T_2 plants 11
Table S8. Analysis of mutations at the *GL2* target site in T-DNA-free T_2 plants 12
Table S9. Heritable mutations at the *TT4* target site in T-DNA-free T_2 plants 14
Table S10. Heritable mutations at the *GL1-1* and *GL1-2* target sites in T-DNA-free T_2 plants 15
Table S11. Analysis of mutations at the *GL1-1* target site in T-DNA-free T_2 plants 16
Table S12. Analysis of mutations at the *GL1-2* target site in T-DNA-free T_2 plants 18
Table S13. Analysis of off-target mutations 20
Table S14. Primer sequences used in this study 21
Table S15. Sequences of target sites 23
Table S16. List of Cas12a vectors generated in this study 24

Fig. S1. RFLP analysis of mutations at the *ECA3-1* target site

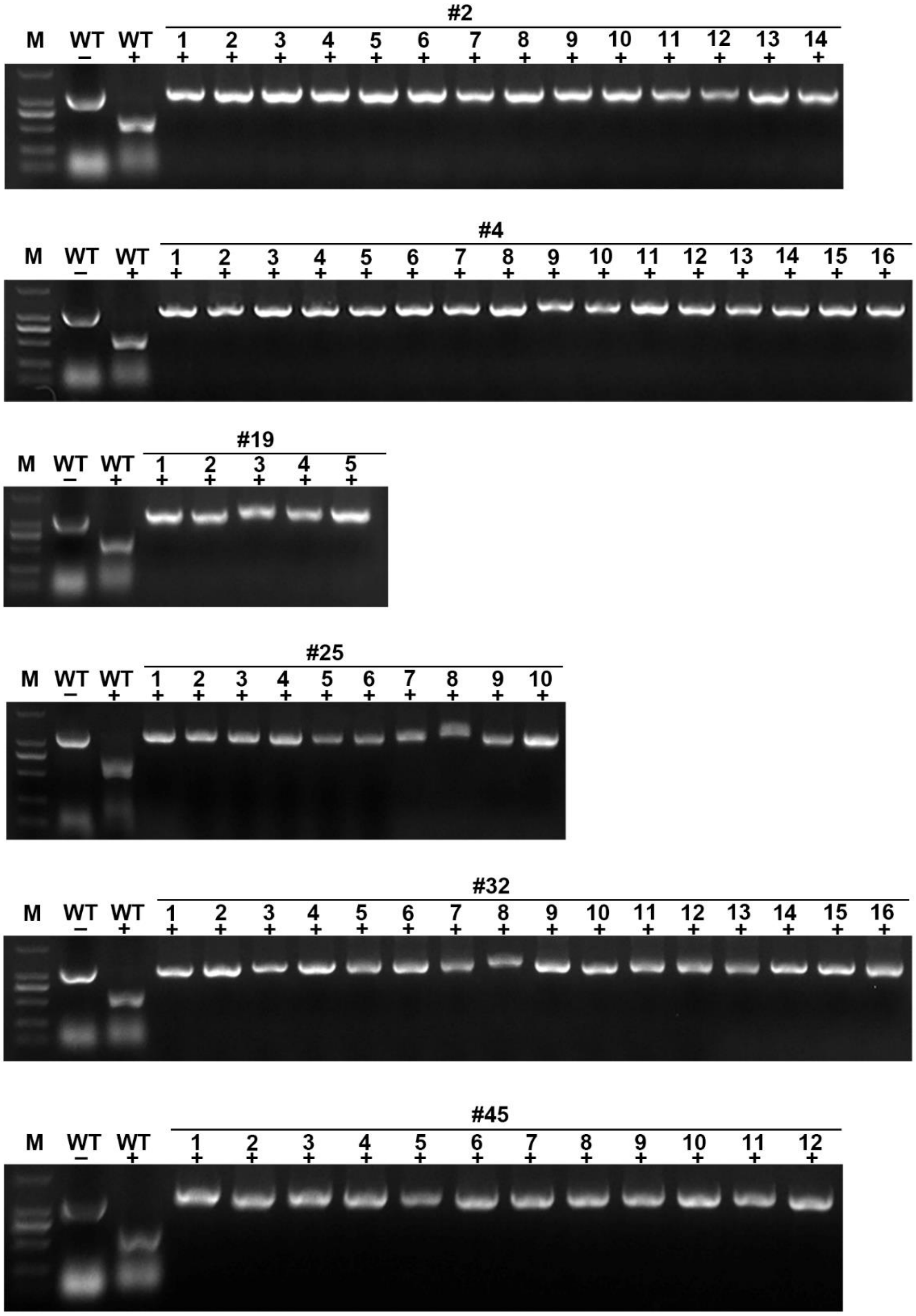

Fig. S1 RFLP analysis of mutations at the *ECA3-1* target site in T-DNA-free T_2 plants derived from T1 plants harboring *ttLbCas12a Ultra* and the U6-tRNA cassette.

PCR products were digested with restriction enzyme *Bgl*II. M, marker; –, undigested PCR products; +, digested PCR products.

Fig. S2. RFLP analysis of mutations at the *ECA3-2* target site

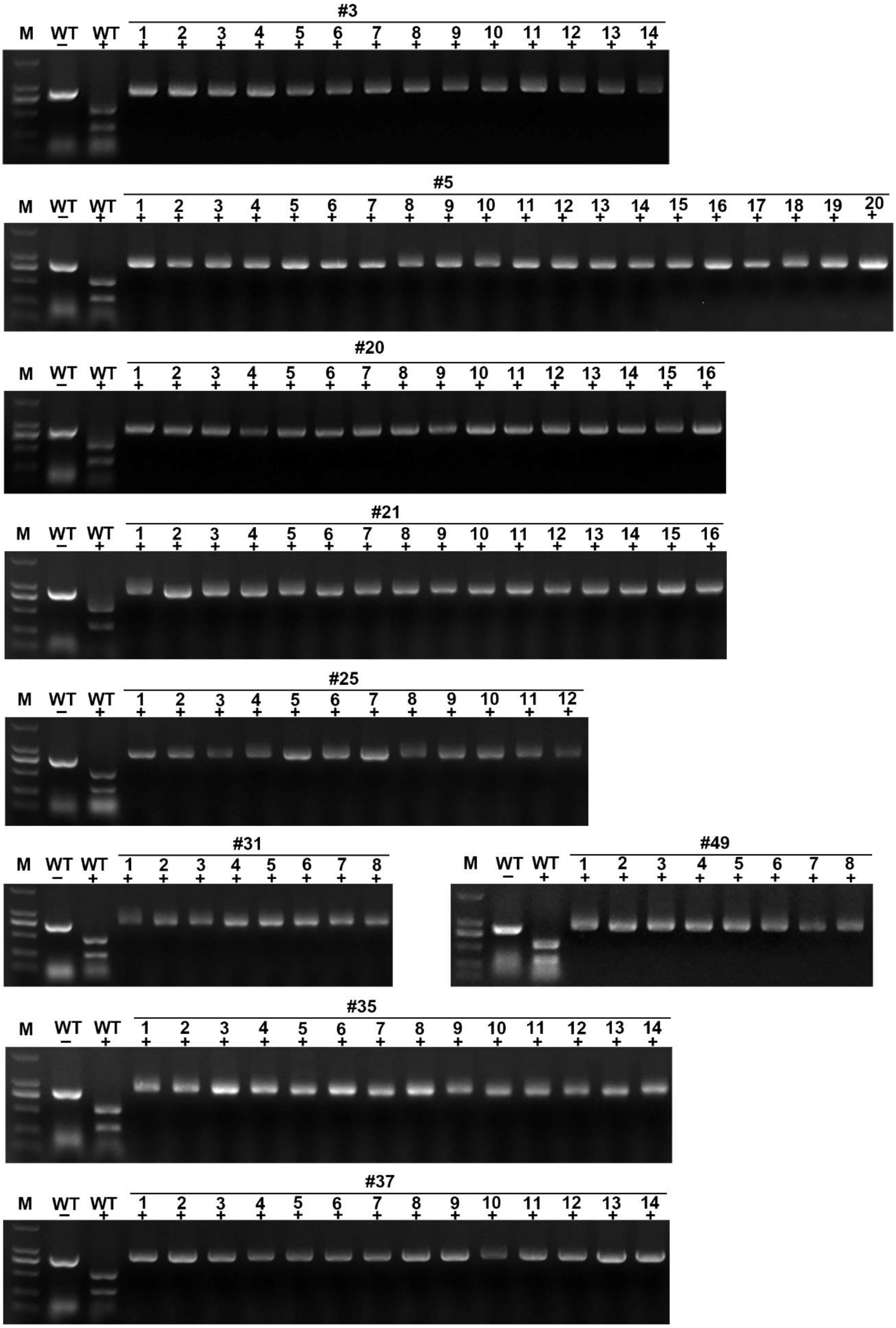

Fig. S2 RFLP analysis of mutations at the *ECA3-2* target site in T-DNA-free T_2 plants derived from T_1 plants harboring ttLbCas12a Ultra and the U6-tRNA cassette.

PCR products were digested with restriction enzyme *Eco*RV. M, marker; –, undigested PCR products; +, digested PCR products.

Table S1. Mutation efficiency of the five Cas12a variants at four target sites for three genes

Table S1. Mutation efficiency of the five Cas12a variants at four target sites for three genes

Target	Cas12a variant	Ratio of Ho/Bi	Ratio of He	Ratio of Chi	Total ratio
ECA3-1	LbCas12a	0.0% (0/77)	0.0% (0/77)	2.6% (2/77)	2.6% (2/77)
	ttLbCas12a	0.0% (0/81)	4.9% (4/81)	1.2% (1/81)	6.2% (5/81)
	ttLbCas12a Ultra	8.0% (7/87)	10.3% (9/87)	27.6% (24/87)	46.0% (40/87)
	AsCas12a Ultra	0.0% (0/70)	0.0% (0/70)	1.4% (1/70)	1.4% (1/70)
	ttAsCas12a Ultra	1.2% (1/86)	1.2% (1/86)	9.3% (8/86)	11.6% (10/86)
ECA3-2	LbCas12a	0.0% (0/95)	3.2% (3/95)	24.2% (23/95)	27.4% (26/95)
	ttLbCas12a	25.0% (24/96)	12.5% (12/96)	43.8% (42/96)	81.3% (78/96)
	ttLbCas12a Ultra	30.5% (29/95)	21.1% (20/95)	33.7% (32/95)	85.3% (81/95)
	AsCas12a Ultra	0.0% (0/95)	1.1% (1/95)	2.1% (2/95)	3.2% (3/95)
	ttAsCas12a Ultra	0.0% (0/96)	5.2% (5/96)	19.8% (19/96)	25.0% (24/96)
GL2	LbCas12a	0.0% (0/96)	0.0% (0/96)	2.1% (2/96)	2.1% (2/96)
	ttLbCas12a	0.0% (0/96)	0.0% (0/96)	2.1% (2/96)	2.1% (2/96)
	ttLbCas12a Ultra	1.0% (1/96)	0.0% (0/96)	14.6% (14/96)	15.6% (15/96)
	AsCas12a Ultra	0.0% (0/96)	0.0% (0/96)	0.0% (0/96)	0.0% (0/96)
	ttAsCas12a Ultra	0.0% (0/96)	0.0% (0/96)	8.3% (8/96)	8.3% (8/96)
TT4	LbCas12a	0.0% (0/95)	0.0% (0/95)	0.0% (0/95)	0.0% (0/95)
	ttLbCas12a	1.1% (1/95)	2.1% (2/95)	14.7% (14/95)	17.9% (17/95)
	ttLbCas12a Ultra	9.6% (9/94)	9.6% (9/94)	21.3% (20/94)	40.4% (38/94)
	AsCas12a Ultra	0.0% (0/96)	0.0% (0/96)	1.0% (1/96)	1.0% (1/96)
	ttAsCas12a Ultra	0.0% (0/95)	1.1% (1/95)	5.3% (5/95)	6.3% (6/95)

The mutation efficiency was calculated based on the ratio between the number of plants with mutations to the total number of transgenic plants. When ≥ 95% high-throughput sequencing reads from a line presented the same type of mutation, this line was scored as a homozygous (Ho) mutant; When ≥ 95% high-throughput sequencing reads from a given line presented more than one type of mutation, this line was considered biallelic (Bi). For non-homozygous and non-biallelic mutants, when ≥ 45% or < 45% high-throughput sequencing reads from a line presented the same type of mutation, these lines were scored as heterozygous (He) or chimeric (Chi) mutants, respectively.

Table S2. Mutation efficiency of the five Cas12a variants at the *GL1-1* and *GL1-2* target sites

Table S2. Mutation efficiency of the five Cas12a variants at the *GL1-1* and *GL1-2* target sites

Target	Cas12a variant	Ratio of Ho/Bi	Ratio of Chi	Total ratio
GL1-1	LbCas12a	18.8% (75/398)	12.6% (50/398)	31.4% (125/398)
	ttLbCas12a	81.2% (233/287)	8.0% (23/287)	89.2% (256/287)
	ttLbCas12a Ultra	86.2% (326/378)	2.9% (11/378)	89.2% (337/378)
	AsCas12a Ultra	7.3% (28/382)	6.8% (26/382)	14.1% (54/382)
	ttAsCas12a Ultra	78.5% (241/307)	16.3% (50/307)	94.8% (291/307)
GL1-2	LbCas12a	2.2% (9/416)	3.1% (13/416)	5.3% (22/416)
	ttLbCas12a	33.8% (98/290)	22.8% (66/290)	56.6% (164/290)
	ttLbCas12a Ultra	49.6% (138/278)	22.7% (63/278)	72.3% (201/278)
	AsCas12a Ultra	0.0% (0/294)	0.0% (0/294)	0.0% (0/294)
	ttAsCas12a Ultra	0.0% (0/250)	0.0% (0/250)	0.0% (0/250)

Mutations in each line was determined by the completely or partially glabrous phenotypes and the mutation efficiency was calculated by the ratio of the number of phenotypically homozygous (Ho), biallelic (Bi), or chimeric (Chi) mutant plants to the total number of transgenic plants.

Table S3. Mutation efficiency of three U6 cassettes

Table S3. Mutation efficiency of three U6 cassettes used to edit the *ECA3-1*, *ECA3-2*, *GL2*, and *TT4* target sites

Target	U6 cassette	Ratio of Ho/Bi	Ratio of He	Ratio of Chi	Total ratio
ECA3-1	U6-tGly	0.0% (0/81)	4.9% (4/81)	1.2% (1/81)	6.2% (5/81)
	U6-HH	1.1% (1/95)	1.1% (1/95)	18.9% (18/95)	21.1% (20/95)
	U6	1.4% (1/72)	1.4% (1/72)	6.9% (5/72)	9.7% (7/72)
ECA3-2	U6-tGly	25.0% (24/96)	12.5% (12/96)	43.8% (42/96)	81.3% (78/96)
	U6-HH	19.8% (19/96)	11.5% (11/96)	41.7% (40/96)	72.9% (70/96)
	U6	35.4% (34/96)	18.8% (18/96)	43.8% (42/96)	97.9% (94/96)
GL2	U6-tGly	0.0% (0/96)	0.0% (0/96)	2.1% (2/96)	2.1% (2/96)
	U6-HH	0.0% (0/93)	0.0% (0/93)	11.8% (11/93)	11.8% (11/93)
	U6	0.0% (0/87)	0.0% (0/87)	6.9% (6/87)	6.9% (6/87)
TT4	U6-tGly	1.1% (1/95)	2.1% (2/95)	14.7% (14/95)	17.9% (17/95)
	U6-HH	1.1% (1/94)	3.2% (3/94)	5.3% (5/94)	9.6% (9/94)
	U6	1.1% (1/90)	0.0% (0/90)	10.0% (9/90)	11.1% (10/90)

The mutation efficiency was calculated based on the ratio between the number of plants with mutations to the total number of transgenic plants. When ≥ 95% high-throughput sequencing reads from a line presented the same type of mutation, this line was scored as a homozygous (Ho) mutant; When ≥ 95% high-throughput sequencing reads from a given line presented more than one type of mutation, this line was considered biallelic (Bi). For non-homozygous and non-biallelic mutants, when ≥ 45% or < 45% high-throughput sequencing reads from a line presented the same type of mutation, these lines were scored as heterozygous (He) or chimeric (Chi) mutants, respectively. The data set from U6-tGly is the same as that in Table S1.

Table S4. Mutation efficiency of the three U6 cassettes at the *GL1-1* and *GL1-2* target sites

Table S4. Mutation efficiency of the three U6 cassettes at the *GL1-1* and *GL1-2* target sites

Target	U6 cassette	Ratio of Ho/Bi	Ratio of Chi	Total ratio
GL1-1	U6-tGly	81.2% (233/287)	8.0% (23/287)	89.2% (256/287)
	U6-HH	54.5% (145/266)	31.6% (84/266)	86.1% (229/266)
	U6	86.6% (291/336)	8.6% (29/336)	95.2% (320/336)
GL1-2	U6-tGly	33.8% (98/290)	22.8% (66/290)	56.6% (164/290)
	U6-HH	8.3% (15/181)	19.3% (35/181)	27.6% (50/181)
	U6	35.1% (52/148)	42.6% (63/148)	77.7% (115/148)

Mutations in each line was determined by the completely or partially glabrous phenotypes and the mutation efficiency was calculated by the ratio of the number of phenotypically homozygous (Ho), biallelic (Bi), or chimeric (Chi) mutant plants to the total number of transgenic plants. The data set from U6-tGly is the same as that in Table S2.

Table S5. Mutation efficiency of different promoters driving the expression of the crRNA

Table S5. Mutation efficiency of different promoters driving the expression of the crRNA designed for the *GL1-2* target site

Target	Promoter	Ratio of Ho/Bi	Ratio of Chi	Total ratio
GL1-2	RPS5A-HH	50.6% (162/320)	24.4% (78/320)	75.0% (240/320)
	UBQ1-HH	61.6% (260/422)	22.5% (95/422)	84.1% (355/422)
	RPS5A-U6-tGly	46.7% (84/180)	36.1% (65/180)	82.8% (149/180)
	UBQ1-U6-tGly	61.5% (80/130)	30.8% (40/130)	92.3% (120/130)
	U6-tGly	49.6% (138/278)	22.7% (63/278)	72.3% (201/278)
	U6	84.9% (242/285)	10.5% (30/285)	95.4% (272/285)

Mutations in each line was determined by the completely or partially glabrous phenotypes and the mutation efficiency was calculated by the ratio of the number of phenotypically homozygous (Ho), biallelic (Bi), or chimeric (Chi) mutant plants to the total number of transgenic plants. The data from U6-tGly is the same as that in Table S2.

Table S6. Mutation efficiency of the two versions of *ttLbCas12a Ultra*

Table S6. Mutation efficiency of the two versions of ttLbCas12a Ultra

Target	Cas12a variant	Ratio of Ho/Bi	Ratio of He	Ratio of Chi	Total ratio
ECA3-1	ttLbCas12a Ultra	1.1% (1/92)	19.6% (18/92)	53.3% (49/92)	73.9% (68/92)
	ttLbCas12a Ultra V2	86.4% (76/88)	11.4% (10/88)	1.1% (1/88)	98.9% (87/88)
GL2	ttLbCas12a Ultra	0.0% (0/137)	1.5% (2/137)	40.9% (56/137)	42.3% (58/137)
	ttLbCas12a Ultra V2	21.3% (30/141)	17.0% (24/141)	60.3% (85/141)	98.6% (139/141)
TT4	ttLbCas12a Ultra	8.6% (8/93)	18.3% (17/93)	51.6% (48/93)	78.5% (73/93)
	ttLbCas12a Ultra V2	86.2% (81/94)	9.6% (9/94)	4.3% (4/94)	100.0% (94/94)
GL1-2	ttLbCas12a Ultra	84.9% (242/285)	/	10.5% (30/285)	95.4% (272/285)
	ttLbCas12a Ultra V2	98.9% (275/278)	/	1.1% (3/278)	100.0% (278/278)

The mutation efficiency was calculated based on the ratio between the number of plants with mutations to the total number of transgenic plants. When ≥ 95% high-throughput sequencing reads from a line presented the same type of mutation, this line was scored as a homozygous (Ho) mutant; When ≥ 95% high-throughput sequencing reads from a given line presented more than one type of mutation, this line was considered biallelic (Bi). For non-homozygous and non-biallelic mutants, when ≥ 45% or < 45% high-throughput sequencing reads from a line presented the same type of mutation, these lines were scored as heterozygous (He) or chimeric (Chi) mutants, respectively. Mutations in GL1-2 in each line was determined by the completely or partially glabrous phenotypes and the mutation efficiency was calculated by the ratio of the number of phenotypically homozygous (Ho), biallelic (Bi), or chimeric (Chi) mutant plants to the total number of transgenic plants. The data of GL1-2 with ttLbCas12a Ultra is the same as that of GL1-2 with U6 in Table S5.

Table S7. Heritable mutations at the *ECA3-1*, *ECA3-2*, and *GL2* target sites in T-DNA-free T_2 plants

Table S7. Heritable mutations at the *ECA3-1*, *ECA3-2*, and *GL2* target sites in T-DNA-free T_2 plants

Target	Line	Mutations in T1	Ratio of T2 mutants
ECA3-1	#2	Bi	100% (14/14)
	#4	Bi	100% (16/16)
	#19	Bi	100% (5/5)
	#25	Bi	100% (10/10)
	#32	Bi	100% (16/16)
	#45	Bi	100% (12/12)
ECA3-2	#3	Bi	100% (14/14)
	#5	Bi	100% (20/20)
	#20	Bi	100% (16/16)
	#21	Bi	100% (16/16)
	#25	Bi	100% (12/12)
	#31	Bi	100% (8/8)
	#35	Bi	100% (14/14)
	#37	Bi	100% (14/14)
	#49	Bi	100% (8/8)
GL2	#32	Bi	100% (30/30)

These T-DNA-free T2 plants were derived from T1 plants harboring ttLbCas12a Ultra and the U6-tRNA cassette.

Table S8. Analysis of mutations at the *GL2* target site in T-DNA-free T_2 plants

Table S8. Analysis of mutations at the *GL2* target site in T-DNA-free T_2 plants

Line	Indel mutations	Indel size	Genotype
#32-1	GATTTGTATGTCAATGGCCGTCGACATGTCTTCCAAACAACCCA (WT)<br>GATTTGTATGTCAATGGCC--------GTCTTCCAAACAACCCA (Allele 1)<br>GATTTGTATGTCAATGGCCGT+ GTCTTCCAAACAACCCA (Allele 2)<br>(+21 bp: TGACTTTGGAGAGGAGAAAAA)	-8 bp<br>+21 bp	Bi
#32-2	GATTTGTATGTCAATGGCC--------GTCTTCCAAACAACCCA (Allele 1)<br>GATTTGTATGTCAATGGCCGT+ GTCTTCCAAACAACCCA (Allele 2)<br>(+21 bp: TGACTTTGGAGAGGAGAAAAA)	-8 bp<br>+21 bp	Bi
#32-3	GATTTGTATGTCAATGGCC--------GTCTTCCAAACAACCCA (Allele 1)<br>GATTTGTATGTCAATGGCCGT+ GTCTTCCAAACAACCCA (Allele 2)<br>(+21 bp: TGACTTTGGAGAGGAGAAAAA)	-8 bp<br>+21 bp	Bi
#32-4	GATTTGTATGTCAATGGCC--------GTCTTCCAAACAACCCA (Allele 1)<br>GATTTGTATGTCAATGGCCGT+ GTCTTCCAAACAACCCA (Allele 2)<br>(+21 bp: TGACTTTGGAGAGGAGAAAAA)	-8 bp<br>+21 bp	Bi
#32-5	GATTTGTATGTCAATGGCC--------GTCTTCCAAACAACCCA (Allele 1)<br>GATTTGTATGTCAATGGCCGT+ GTCTTCCAAACAACCCA (Allele 2)<br>(+21 bp: TGACTTTGGAGAGGAGAAAAA)	-8 bp<br>+21 bp	Bi
#32-6	GATTTGTATGTCAATGGCC--------GTCTTCCAAACAACCCA (Allele 1)<br>GATTTGTATGTCAATGGCCGT+ GTCTTCCAAACAACCCA (Allele 2)<br>(+21 bp: TGACTTTGGAGAGGAGAAAAA)	-8 bp<br>+21 bp	Bi
#32-7	GATTTGTATGTCAATGGCC--------GTCTTCCAAACAACCCA (Allele 1)<br>GATTTGTATGTCAATGGCC--------GTCTTCCAAACAACCCA (Allele 2)	-8 bp<br>-8 bp	Ho
#32-8	GATTTGTATGTCAATGGCC--------GTCTTCCAAACAACCCA (Allele 1)<br>GATTTGTATGTCAATGGCCGT+ GTCTTCCAAACAACCCA (Allele 2)<br>(+21 bp: TGACTTTGGAGAGGAGAAAAA)	-8 bp<br>+21 bp	Bi
#32-9	GATTTGTATGTCAATGGCC--------GTCTTCCAAACAACCCA (Allele 1)<br>GATTTGTATGTCAATGGCCGT+ GTCTTCCAAACAACCCA (Allele 2)<br>(+21 bp: TGACTTTGGAGAGGAGAAAAA)	-8 bp<br>+21 bp	Bi
#32-10	GATTTGTATGTCAATGGCC--------GTCTTCCAAACAACCCA (Allele 1)<br>GATTTGTATGTCAATGGCCGT+ GTCTTCCAAACAACCCA (Allele 2)<br>(+21 bp: TGACTTTGGAGAGGAGAAAAA)	-8 bp<br>+21 bp	Bi
#32-11	GATTTGTATGTCAATGGCCGT+ GTCTTCCAAACAACCCA (Allele 1)<br>GATTTGTATGTCAATGGCCGT+ GTCTTCCAAACAACCCA (Allele 2)<br>(+21 bp: TGACTTTGGAGAGGAGAAAAA)	+21 bp<br>+21 bp	Ho
#32-12	GATTTGTATGTCAATGGCC--------GTCTTCCAAACAACCCA (Allele 1)<br>GATTTGTATGTCAATGGCCGT+ GTCTTCCAAACAACCCA (Allele 2)<br>(+21 bp: TGACTTTGGAGAGGAGAAAAA)	-8 bp<br>+21 bp	Bi
#32-13	GATTTGTATGTCAATGGCC--------GTCTTCCAAACAACCCA (Allele 1)<br>GATTTGTATGTCAATGGCCGT+ GTCTTCCAAACAACCCA (Allele 2)<br>(+21 bp: TGACTTTGGAGAGGAGAAAAA)	-8 bp<br>+21 bp	Bi
#32-14	GATTTGTATGTCAATGGCC--------GTCTTCCAAACAACCCA (Allele 1)<br>GATTTGTATGTCAATGGCCGT+ GTCTTCCAAACAACCCA (Allele 2)<br>(+21 bp: TGACTTTGGAGAGGAGAAAAA)	-8 bp<br>+21 bp	Bi
#32-15	GATTTGTATGTCAATGGCCGT+ GTCTTCCAAACAACCCA (Allele 1)<br>GATTTGTATGTCAATGGCCGT+ GTCTTCCAAACAACCCA (Allele 2)<br>(+21 bp: TGACTTTGGAGAGGAGAAAAA)	+21 bp<br>+21 bp	Ho
#32-16	GATTTGTATGTCAATGGCC--------GTCTTCCAAACAACCCA (Allele 1)<br>GATTTGTATGTCAATGGCCGT+ GTCTTCCAAACAACCCA (Allele 2)<br>(+21 bp: TGACTTTGGAGAGGAGAAAAA)	-8 bp<br>+21 bp	Bi
#32-17	GATTTGTATGTCAATGGCCGT+ GTCTTCCAAACAACCCA (Allele 1)<br>GATTTGTATGTCAATGGCCGT+ GTCTTCCAAACAACCCA (Allele 2)<br>(+21 bp: TGACTTTGGAGAGGAGAAAAA)	+21 bp<br>+21 bp	Ho
#32-18	GATTTGTATGTCAATGGCC--------GTCTTCCAAACAACCCA (Allele 1)<br>GATTTGTATGTCAATGGCCGT+ GTCTTCCAAACAACCCA (Allele 2)<br>(+21 bp: TGACTTTGGAGAGGAGAAAAA)	-8 bp<br>+21 bp	Bi
#32-19	GATTTGTATGTCAATGGCC--------GTCTTCCAAACAACCCA (Allele 1)<br>GATTTGTATGTCAATGGCC--------GTCTTCCAAACAACCCA (Allele 2)	-8 bp<br>-8 bp	Ho

#32-20	GATTTGTATGTCAATGGCC--------GTCTTCCAAACAACCCA (Allele 1)<br>GATTTGTATGTCAATGGCCGT+ GTCTTCCAAACAACCCA (Allele 2)<br>(+21 bp: TGACTTTGGAGAGGAGAAAAA)	-8 bp<br>+21 bp	Bi
#32-21	GATTTGTATGTCAATGGCC--------GTCTTCCAAACAACCCA (Allele 1)<br>GATTTGTATGTCAATGGCCGT+ GTCTTCCAAACAACCCA (Allele 2)<br>(+21 bp: TGACTTTGGAGAGGAGAAAAA)	-8 bp<br>+21 bp	Bi
#32-22	GATTTGTATGTCAATGGCC--------GTCTTCCAAACAACCCA (Allele 1)<br>GATTTGTATGTCAATGGCC--------GTCTTCCAAACAACCCA (Allele 2)	-8 bp<br>-8 bp	Ho
#32-23	GATTTGTATGTCAATGGCCGT+ GTCTTCCAAACAACCCA (Allele 1)<br>GATTTGTATGTCAATGGCCGT+ GTCTTCCAAACAACCCA (Allele 2)<br>(+21 bp: TGACTTTGGAGAGGAGAAAAA)	+21 bp<br>+21 bp	Ho
#32-24	GATTTGTATGTCAATGGCC--------GTCTTCCAAACAACCCA (Allele 1)<br>GATTTGTATGTCAATGGCC--------GTCTTCCAAACAACCCA (Allele 2)	-8 bp<br>-8 bp	Ho
#32-25	GATTTGTATGTCAATGGCC--------GTCTTCCAAACAACCCA (Allele 1)<br>GATTTGTATGTCAATGGCC--------GTCTTCCAAACAACCCA (Allele 2)	-8 bp<br>-8 bp	Ho
#32-26	GATTTGTATGTCAATGGCCGT+ GTCTTCCAAACAACCCA (Allele 1)<br>GATTTGTATGTCAATGGCCGT+ GTCTTCCAAACAACCCA (Allele 2)<br>(+21 bp: TGACTTTGGAGAGGAGAAAAA)	+21 bp<br>+21 bp	Ho
#32-27	GATTTGTATGTCAATGGCC--------GTCTTCCAAACAACCCA (Allele 1)<br>GATTTGTATGTCAATGGCCGT+ GTCTTCCAAACAACCCA (Allele 2)<br>(+21 bp: TGACTTTGGAGAGGAGAAAAA)	-8 bp<br>+21 bp	Bi
#32-28	GATTTGTATGTCAATGGCCGT+ GTCTTCCAAACAACCCA (Allele 1)<br>GATTTGTATGTCAATGGCCGT+ GTCTTCCAAACAACCCA (Allele 2)<br>(+21 bp: TGACTTTGGAGAGGAGAAAAA)	+21 bp<br>+21 bp	Ho
#32-29	GATTTGTATGTCAATGGCC--------GTCTTCCAAACAACCCA (Allele 1)<br>GATTTGTATGTCAATGGCCGT+ GTCTTCCAAACAACCCA (Allele 2)<br>(+21 bp: TGACTTTGGAGAGGAGAAAAA)	-8 bp<br>+21 bp	Bi
#32-30	GATTTGTATGTCAATGGCC--------GTCTTCCAAACAACCCA (Allele 1)<br>GATTTGTATGTCAATGGCC--------GTCTTCCAAACAACCCA (Allele 2)	-8 bp<br>-8 bp	Ho

These T-DNA-free T2 plants were derived from a T1 biallelic mutant plant harboring ttLbCas12a Ultra and the U6-tRNA cassette. Red letters indicate PAM, blue letters indicate spacers, and yellow letters indicate insertion.

Table S9. Heritable mutations at the *TT4* target site in T-DNA-free T_2 plants

Table S9. Heritable mutations at the *TT4* target site in T-DNA-free T_2 plants

Target	Line	Mutations in T1	Ratio of T2 mutants
TT4	#4	Ho	100% (5/5)
	#5	Bi	100% (11/11)
	#10	Bi	100% (45/45)
	#13	Bi	100% (30/30)
	#14	Bi	100% (12/12)
	#22	Bi	100% (27/27)
	#31	Bi	100% (22/22)

These T-DNA-free T2 plants were derived from T1 plants harboring ttLbCas12a Ultra and the U6-tRNA cassette.

Table S10. Heritable mutations at the *GL1-1* and *GL1-2* target sites in T-DNA-free T_2 plants

Table S10. Heritable mutations at the *GL1-1* and *GL1-2* target sites in T-DNA-free T_2 plants

Target	Line	Mutations in T1	Ratio of T2 mutants
GL1-1	#11	Phenotypically Ho/Bi	100% (37/37)
	#13	Phenotypically Ho/Bi	100% (47/47)
	#22	Phenotypically Ho/Bi	100% (50/50)
	#23	Phenotypically Ho/Bi	100% (48/48)
	#25	Phenotypically Ho/Bi	100% (45/45)
	#28	Phenotypically Ho/Bi	100% (43/43)
	#31	Phenotypically Ho/Bi	100% (49/49)
	#36	Phenotypically Ho/Bi	100% (51/51)
	#38	Phenotypically Ho/Bi	100% (37/37)
	#43	Phenotypically Ho/Bi	100% (49/49)
GL1-2	#8	Phenotypically Ho/Bi	100% (37/37)
	#23	Phenotypically Ho/Bi	100% (48/48)
	#36	Phenotypically Ho/Bi	100% (47/47)
	#55	Phenotypically Ho/Bi	100% (48/48)
	#56	Phenotypically Ho/Bi	100% (45/45)

These T-DNA-free T2 plants were derived from T1 plants harboring ttLbCas12a Ultra and the U6-tRNA cassette.

Table S11. Analysis of mutations at the *GL1-1* target site in T-DNA-free T_2 plants

Table S11. Analysis of mutations at the *GL1-1* target site in T-DNA-free T_2 plants

Line	Indel mutations	Indel size	Genotype
#11-3	AATTTCACTGAACAAGAAGAAGACCTCATTATTCGTCTCCACAA (WT)<br>AATTTCACTGAACAAGAAGA----------------CTCCACAA (Allel 1)<br>AATTTCACTGAACAAGAAGA----------------CTCCACAA (Allel 2)	-16 bp<br>-16 bp	Ho
#11-4	AATTTCACTGAACAAGAAGAA-------TTATTCGTCTCCACAA (Allel 1)<br>AATTTCACTGAACAAGAAGAA-------TTATTCGTCTCCACAA (Allel 2)	-7 bp<br>-7 bp	Ho
#11-5	AATTTCACTGAACAAGAAGA----------------CTCCACAA (Allel 1)<br>AATTTCACTGAACAAGAAGA----------------CTCCACAA (Allel 2)	-16 bp<br>-16 bp	Ho
#11-6	AATTTCACTGAACAAGAAGA----------------CTCCACAA (Allel 1)<br>AATTTCACTGAACAAGAAGA----------------CTCCACAA (Allel 2)	-16 bp<br>-16 bp	Ho
#13-1	AATTTCACTGAACAAGAAGA---------TATTCGTCTCCACAA (Allel 1)<br>AATTTCACTGAACAAGAAGA---------TATTCGTCTCCACAA (Allel 2)	-9 bp<br>-9 bp	Ho
#13-2	AATTTCACTGAACAAGAAGA---------TATTCGTCTCCACAA (Allel 1)<br>AATTTCACTGAACAAGAAGAAG-------TATTCGTCTCCACAA (Allel 2)	-9 bp<br>-7 bp	Bi
#13-3	AATTTCACTGAACAAGAAGA---------TATTCGTCTCCACAA (Allel 1)<br>AATTTCACTGAACAAGAAGAAG-------TATTCGTCTCCACAA (Allel 2)	-9 bp<br>-7 bp	Bi
#13-4	AATTTCACTGAACAAGAAGA---------TATTCGTCTCCACAA (Allel 1)<br>AATTTCACTGAACAAGAAGAAG-------TATTCGTCTCCACAA (Allel 2)	-9 bp<br>-7 bp	Bi
#22-1	AATTTCACTGAACAAGAAGAA-------TTATTCGTCTCCACAA (Allel 1)<br>AATTTCACTGAACAAGAAGAA-------TTATTCGTCTCCACAA (Allel 2)	-7 bp<br>-7 bp	Ho
#22-2	AATTTCACTGAACAAGAAGAA-------TTATTCGTCTCCACAA (Allel 1)<br>AATTTCACTGAACAAGAAGAA-------TTATTCGTCTCCACAA (Allel 2)	-7 bp<br>-7 bp	Ho
#22-3	AATTTCACTGAACAAGAAGAA-------TTATTCGTCTCCACAA (Allel 1)<br>AATTTCACTGAACAAGAAGAA-------TTATTCGTCTCCACAA (Allel 2)	-7 bp<br>-7 bp	Ho
#22-5	AATTTCACTGAACAAGAAGAA-------TTATTCGTCTCCACAA (Allel 1)<br>AATTTCACTGAACAAGAAGAA-------TTATTCGTCTCCACAA (Allel 2)	-7 bp<br>-7 bp	Ho
#22-6	AATTTCACTGAACAAGAAGAA-------TTATTCGTCTCCACAA (Allel 1)<br>AATTTCACTGAACAAGAAGAA-------TTATTCGTCTCCACAA (Allel 2)	-7 bp<br>-7 bp	Ho
#23-1	AATTTCACTGAA----------------TTATTCGTCTCCACAA (Allel 1)<br>AATTTCACTGAA----------------TTATTCGTCTCCACAA (Allel 2)	-16 bp<br>-16 bp	Ho
#23-2	AATTTCACTGAA----------------TTATTCGTCTCCACAA (Allel 1)<br>AATTTCACTGAACAAGAAGAA-------TTATTCGTCTCCACAA (Allel 2)	-16 bp<br>-7 bp	Bi
#23-3	AATTTCACTGAA----------------TTATTCGTCTCCACAA (Allel 1)<br>AATTTCACTGAACAAGAAGAA-------TTATTCGTCTCCACAA (Allel 2)	-16 bp<br>-7 bp	Bi
#23-4	AATTTCACTGAACAAGAAGAAGAC-----TATTCGTCTCCACAA (Allel 1)<br>AATTTCACTGAACAAGAAGAAGAC-----TATTCGTCTCCACAA (Allel 2)	-5 bp<br>-5 bp	Ho
#25-1	AATTTCACTGAACAAGAAGAAG---------TTCGTCTCCACAA (Allel 1)<br>AATTTCACTGAACAAGAAGAAGA---+ATTATTCGTCTCCACAA (Allel 2)<br>(+3 bp: TGA)	-9 bp<br>-4&+3 bp	Bi
#25-2	AATTTCACTGAACAAGAAGAAG---------TTCGTCTCCACAA (Allel 1)<br>AATTTCACTGAACAAGAAGAAGA---+ATTATTCGTCTCCACAA (Allel 2)<br>(+3 bp: TGA)	-9 bp<br>-4&+3 bp	Bi
#25-3	AATTTCACTGAACAAGAAGAAGA---+ATTATTCGTCTCCACAA (Allel 1)<br>AATTTCACTGAACAAGAAGAAGA---+ATTATTCGTCTCCACAA (Allel 2)<br>(+3 bp: TGA)	-4&+3 bp<br>-4&+3 bp	Ho
#25-4	AATTTCACTGAACAAGAAGAAGA---+ATTATTCGTCTCCACAA (Allel 1)<br>AATTTCACTGAACAAGAAGAAGA---+ATTATTCGTCTCCACAA (Allel 2)<br>(+3 bp: TGA)	-4&+3 bp<br>-4&+3 bp	Ho
#28-1	AATTTCACTGAACAAGAAGAA-------TTATTCGTCTCCACAA (Allel 1)<br>AATTTCACTGAACAAGAAGAAGA-----------+TCTCCACAA (Allel 2)<br>(+10 bp:TCATTATCCC)	-7 bp<br>-12&+10 bp	Bi
#28-2	AATTTCACTGAACAAGAAGAA-------TTATTCGTCTCCACAA (Allel 1)<br>AATTTCACTGAACAAGAAGAA-------TTATTCGTCTCCACAA (Allel 2)	-7 bp<br>-7 bp	Ho
#28-3	AATTTCACTGAACAAGAAGAA-------TTATTCGTCTCCACAA (Allel 1)<br>AATTTCACTGAACAAGAAGAAGA--TCATTATTCGTCTCCACAA (Allel 2)	-7 bp<br>-2 bp	Bi
#28-4	AATTTCACTGAACAAGAAGAA-------TTATTCGTCTCCACAA (Allel 1)<br>AATTTCACTGAACAAGAAGAA-------TTATTCGTCTCCACAA (Allel 2)	-7 bp<br>-7 bp	Ho
#31-1	AATTTCACTGAACAAGAAGAA-------TTATTCGTCTCCACAA (Allel 1)<br>AATTTCACTGAACAAGAAGAAG------TTATTCGTCTCCACAA (Allel 2)	-7 bp<br>-6 bp	Bi

#31-2	AATTTCACTGAACAAGAAGAA-------TTATTCGTCTCCACAA (Allel 1)<br>AATTTCACTGAACAAGAAGAAG------TTATTCGTCTCCACAA (Allel 2)	-7 bp<br>-6 bp	Bi
#31-3	AATTTCACTGAACAAGAAGAA-------TTATTCGTCTCCACAA (Allel 1)<br>AATTTCACTGAACAAGAAGAAG------TTATTCGTCTCCACAA (Allel 2)	-7 bp<br>-6 bp	Bi
#31-4	AATTTCACTGAACAAGAAGAA-------TTATTCGTCTCCACAA (Allel 1)<br>AATTTCACTGAACAAGAAGAAG------TTATTCGTCTCCACAA (Allel 2)	-7 bp<br>-6 bp	Bi
#31-5	AATTTCACTGAACAAGAAGAA-------TTATTCGTCTCCACAA (Allel 1)<br>AATTTCACTGAACAAGAAGAAG------TTATTCGTCTCCACAA (Allel 2)	-7 bp<br>-6 bp	Bi
#31-6	AATTTCACTGAACAAGAAGAA-------TTATTCGTCTCCACAA (Allel 1)<br>AATTTCACTGAACAAGAAGAA-------TTATTCGTCTCCACAA (Allel 2)	-7 bp<br>-7 bp	Ho
#36-1	AATTTCACTGAACAAGAAG---------TTATTCGTCTCCACAA (Allel 1)<br>AATTTCACTGAACAAGAAG---------TTATTCGTCTCCACAA (Allel 2)	-9 bp<br>-9 bp	Ho
#36-2	AATTTCACTGAACAAGAA----------TTATTCGTCTCCACAA (Allel 1)<br>AATTTCACTGAACAAGAAG---------TTATTCGTCTCCACAA (Allel 2)	-10 bp<br>-9 bp	Bi
#36-3	AATTTCACTGAACAAGAAGAA-------TTATTCGTCTCCACAA (Allel 1)<br>AATTTCACTGAACAAGAAGAA-------TTATTCGTCTCCACAA (Allel 2)	-7 bp<br>-7 bp	Ho
#36-4	AATTTCACTGAACAAGAA----------TTATTCGTCTCCACAA (Allel 1)<br>AATTTCACTGAACAAGAA----------TTATTCGTCTCCACAA (Allel 2)	-10 bp<br>-10 bp	Ho
#38-1	AATTTCACTGAACAAGAAGAA---CTCATTATTCGTCTCCACAA (Allel 1)<br>AATTTCACTGAACAAGAAGAA---CTCATTATTCGTCTCCACAA (Allel 2)	-3 bp<br>-3 bp	Ho
#38-2	AATTTCACTGAACAA+-----------ATTATTCGTCTCCACAA (Allel 1)<br>AATTTCACTGAACAA+-----------ATTATTCGTCTCCACAA (Allel 2)<br>(+24 bp:AGGCAATTTCACTGAGATGGATGA)	-12&+24 bp<br>-12&+24 bp	Ho
#38-3	AATT----------------------------------CCACAA (Allel 1)<br>AATTTCACTGAACAA+-----------ATTATTCGTCTCCACAA (Allel 2)<br>(+24 bp:AGGCAATTTCACTGAGATGGATGA)	-34 bp<br>-12&+24 bp	Bi
#38-4	AATTTCACTGAACAAGAAGAA---CTCATTATTCGTCTCCACAA (Allel 1)<br>AATTTCACTGAACAAGAAGAA---CTCATTATTCGTCTCCACAA (Allel 2)	-3 bp<br>-3 bp	Ho
#38-5	AATTTCAC--------------------------GTCTCCACAA (Allel 1)<br>AATTTCAC--------------------------GTCTCCACAA (Allel 2)	-26 bp<br>-26 bp	Ho
#38-6	AATTTCACTGAACAAGAAGAA---CTCATTATTCGTCTCCACAA (Allel 1)<br>AATTTCACTGAACAAGAAGAA---CTCATTATTCGTCTCCACAA (Allel 2)	-3 bp<br>-3 bp	Ho
#43-1	AATTTCACTGAACAAGAAGAAGAt-TCATTATTCGTCTCCACAA (Allel 1)<br>AATTTCACTGAACAAGAAGAAGAt-TCATTATTCGTCTCCACAA (Allel 2)	-2&+1 bp<br>-2&+1 bp	Ho
#43-2	AATTTCACTGAACAAGAAGAAGAC-----TATTCGTCTCCACAA (Allel 1)<br>AATTTCACTGAACAAGAAGAAGAC-----TATTCGTCTCCACAA (Allel 2)	-5 bp<br>-5 bp	Ho
#43-4	AATTTCACTGAACAAGAAGAAGAC-----TATTCGTCTCCACAA (Allel 1)<br>AATTTCACTGAACAAGAAGAAGAC-----TATTCGTCTCCACAA (Allel 2)	-5 bp<br>-5 bp	Ho
#43-5	AATTTCACTGAACAAGAAGAAG------TTATTCGTCTCCACAA (Allel 1)<br>AATTTCACTGAACAAGAAGAAGAt-TCATTATTCGTCTCCACAA (Allel 2)	-6 bp<br>-2&+1 bp	Bi
#43-6	AATTTCACTGAACAAGAAGAAG------TTATTCGTCTCCACAA (Allel 1)<br>AATTTCACTGAACAAGAAGAAGAt-TCATTATTCGTCTCCACAA (Allel 2)	-6 bp<br>-2&+1 bp	Bi

These T-DNA-free T2 plants were derived from T1 plants harboring ttLbCas12a Ultra and the U6-tRNA cassette. Red letters indicate PAM, blue letters indicate spacers, and yellow letters indicate insertion.

Table S12. Analysis of mutations at the *GL1-2* target site in T-DNA-free T_2 plants

Table S12. Analysis of mutations at the *GL1-2* target site in T-DNA-free T_2 plants

Line	Indel mutations	Indel size	Genotype
#8-1	GGTCTTTGATAGCTAAAAGAGTACCGGGAAGAACAGATAACCAA (WT)<br>GGTCTTTGATAGCTAAAAGAGTACC------------------- (Allel 1)<br>GGTCTTTGATAGCTAAAAGAGTACC------------------- (Allel 2)	-28 bp<br>-28 bp	Ho
#8-2	GGTCTTTGATAGCTAAAAGAGTACC------------------- (Allel 1)<br>GGTCTTTGATAGCTAAAAGAGTAC-----------+ATAACCAA (Allel 2)<br>(+14 bp:TCCTCCGCCGACAA)	-28 bp<br>-12&+14 bp	Bi
#8-3	GGTCTTTGATAGCTAAAAGAGTACC------------------- (Allel 1)<br>GGTCTTTGATAGCTAAAAGAGTACC------------------- (Allel 2)	-28 bp<br>-28 bp	Ho
#8-4	GGTCTTTGATAGCTAAAAGAGTACC------------------- (Allel 1)<br>GGTCTTTGATAGCTAAAAGAGTACC------------------- (Allel 2)	-28 bp<br>-28 bp	Ho
#8-5	GGTCTTTGATAGCTAAAAGAGTACC------------------- (Allel 1)<br>GGTCTTTGATAGCTAAAAGAGTAC-----------+ATAACCAA (Allel 2)<br>(+14 bp:TCCTCCGCCGACAA)	-28 bp<br>-12&+14 bp	Bi
#8-6	GGTCTTTGATAGCTAAAAGAGTACC------------------- (Allel 1)<br>GGTCTTTGATAGCTAAAAGAGTACC------------------- (Allel 2)	-28 bp<br>-28 bp	Ho
#23-1	GGTCTTTGATAGCTAAAAGAGTA---------ACAGATAACCAA (Allel 1)<br>GGTCTTTGATAGCTAAAAGAGTA---------ACAGATAACCAA (Allel 2)	-9 bp<br>-9 bp	Ho
#23-2	GGTCTTTGATAGCTAAAAGAGTA---------ACAGATAACCAA (Allel 1)<br>GGTCTTTGATAGCTAAAAGAGTA---------ACAGATAACCAA (Allel 2)	-9 bp<br>-9 bp	Ho
#23-3	GGTCTTTGATAGCTAAAAGAGTA---------ACAGATAACCAA (Allel 1)<br>GGTCTTTGATAGCTAAAAGAGTA---------ACAGATAACCAA (Allel 2)	-9 bp<br>-9 bp	Ho
#23-4	GGTCTTTGATAGCTAAAAGAGTA---------ACAGATAACCAA (Allel 1)<br>GGTCTTTGATAGCTAAAAGAGTA---------ACAGATAACCAA (Allel 2)	-9 bp<br>-9 bp	Ho
#23-5	GGTCTTTGATAGCTAAAAGAGTA---------ACAGATAACCAA (Allel 1)<br>GGTCTTTGATAGCTAAAAGAGTA---------ACAGATAACCAA (Allel 2)	-9 bp<br>-9 bp	Ho
#23-6	GGTCTTTGATAGCTAAAAGAGTA---------ACAGATAACCAA (Allel 1)<br>GGTCTTTGATAGCTAAAAGAGTA---------ACAGATAACCAA (Allel 2)	-9 bp<br>-9 bp	Ho
#36-1	GGTCTTTGATAGCTAAAAGAGTACCG----------ATAACCAA (Allel 1)<br>GGTCTTTGATAGCTAAAAGAGTACCG----------ATAACCAA (Allel 2)	-10 bp<br>-10 bp	Ho
#36-2	GGTCTTTGATAGCTAAAAGAGTACCG----------ATAACCAA (Allel 1)<br>GGTCTTTGATAGCTAAAAGAGTACCG----------ATAACCAA (Allel 2)	-10 bp<br>-10 bp	Ho
#36-3	GGTCTTTGATAGCTAA---------------------------- (Allel 1)<br>GGTCTTTGATAGCTAAAAGAGTACCG----------ATAACCAA (Allel 2)	-28 bp<br>-10 bp	Bi
#36-4	GGTCTTTGATAGCTAA---------------------------- (Allel 1)<br>GGTCTTTGATAGCTAA---------------------------- (Allel 2)	-28 bp<br>-28 bp	Ho
#36-5	GGTCTTTGATAGCTAA---------------------------- (Allel 1)<br>GGTCTTTGATAGCTAAAAGAGTACCG----------ATAACCAA (Allel 2)	-28 bp<br>-10 bp	Bi
#36-6	GGTCTTTGATAGCTAA---------------------------- (Allel 1)<br>GGTCTTTGATAGCTAAAAGAGTACCG----------ATAACCAA (Allel 2)	-28 bp<br>-10 bp	Bi
#55-1	GGTCTTTGATAGCTAAAAGAGTAC------GAACAGATAACCAA (Allel 1)<br>GGTCTTTGATAGCTAAAAGAGTAC------GAACAGATAACCAA (Allel 2)	-6 bp<br>-6 bp	Ho
#55-2	GGTCTTTGATAGCTAAAAG------------------------- (Allel 1)<br>GGTCTTTGATAGCTAAAAG------------------------- (Allel 2)	-26 bp<br>-26 bp	Ho

#55-3	GGTCTTTGATAGCTAAAAGAGTAC-----AGAACAGATAACCAA (Allel 1)<br>GGTCTTTGATAGCTAAAAGAGTAC-----AGAACAGATAACCAA (Allel 2)	-5 bp<br>-5 bp	Ho
#55-4	GGTCTTTGATAGCTAAAAGAGTAC-----AGAACAGATAACCAA (Allel 1)<br>GGTCTTTGATAGCTAAAAG------------------------- (Allel 2)	-5 bp<br>-26 bp	Bi
#55-5	GGTCTTTGATAGCTAAAAGAGTAC-----AGAACAGATAACCAA (Allel 1)<br>GGTCTTTGATAGCTAAAAG------------------------- (Allel 2)	-5 bp<br>-26 bp	Bi
#55-6	GGTCTTTGATAGCTAAAAGAGTAC-----AGAACAGATAACCAA (Allel 1)<br>GGTCTTTGATAGCTAAAAGAGTAC-----AGAACAGATAACCAA (Allel 2)	-5 bp<br>-5 bp	Ho
#56-1	--CCAA (Allel 1)<br>--CCAA (Allel 2)	-281 bp<br>-281 bp	Ho
#56-2	--CCAA (Allel 1)<br>--CCAA (Allel 2)	-281 bp<br>-281 bp	Ho
#56-3	--CCAA (Allel 1)<br>--CCAA (Allel 2)	-281 bp<br>-281 bp	Ho
#56-4	--CCAA (Allel 1)<br>--CCAA (Allel 2)	-281 bp<br>-281 bp	Ho
#56-5	--CCAA (Allel 1)<br>--CCAA (Allel 2)	-281 bp<br>-281 bp	Ho
#56-6	--CCAA (Allel 1)<br>--CCAA (Allel 2)	-281 bp<br>-281 bp	Ho

These T-DNA-free T2 plants were derived from T1 plants harboring ttLbCas12a Ultra and the U6-tRNA cassette. Red letters indicate PAM, blue letters indicate spacers, and yellow letters indicate insertion.

Table S13. Analysis of off-target mutations

Table S13. Analysis of off-target mutations

Vector	Target and off-target genes	Sequence of target and off-target	No. of mismatches	No. of mutants analyzed	No. of mutants with off-target mutations
pttLbU01-	*GL1*	**TTTC**ACTGAACAAGAAGAAGACCTCAT	/	/	/
GL1-1	*At5G40330*	**TTTt**ACTGAcCAAGAAGAAGAtCTCAT	3	36	0
	At1G22640	**TTTt**ACTGAAgAAGAAGAtGAaCTCAT	4	36	0
pttLbU2-	*GL1*	**TTTG**ATAGCTAAAAGAGTACCGGGAAG	/	/	/
GL1-2	*At5G14750*	**TTTa**ATtGCTAAAAGAGTgCCGGGtcG	5	36	0

PAMs are in boldface and underlined, and mismatched bases are in lower case and red.

Table S14. Primer sequences used in this study

Table S14. Primer sequences used in this study

Name	Sequence	Purpose
oHA-BS-F	GATCCTACCCTTACGACGTGCCAGACTACGCTTGAGCT	Vector construction
oHA-BS-R	CAAGCGTAGTCTGGCACGTCGTAAGGGTAG	
pUbi-IDF2	TTTAGCCCTGCCTTCATACGCTATT	
E795L-BsR	ATTATTATTGGTCTCAGCAGGTACTGGTCCTCGCTGAA	
E795L-BsF	ATTTATTTATTTGGTCTCACTGCTGCACATCCCGAT	
tE9-IDR2	TTCCCAATGCCATAATACTCAAACTC	
As12a-XbF	ACATTATTACCGCTCTAGATGGCTC	
E174R-BsR	ATTATTATTGGTCTCATGCGGTAGAAGCCGGAGAAGTAC	
E174R-BsF	ATTATTTATTTGGTCTCACGCAACCGCAAGAACGTGTTC	
M537R-BsR	ATTATTATTGGTCTCAGCCTCTGGAAGTTGAGCTTGAA	
M537R-BsF	ATTATTTATTTGGTCTCAAGGCCGACCCTC	
F870L-BsR	ATTATTTATTGGTCTCAGAGGAACTTGTCGGAGGTGAA	
F870L-BsF	ATTATTTATTTGGTCTCACCTCTTCCACGTCCCCAT	
As12a-SaR	ATTATTGCCTGAGCTCAAGCGTAGTC	
U6-AvF	ATTTATTTTACCTAGGCGACTTGCCTTCCGCAC	
AsDR-AaR	ATTATTTATTTACACCTGCATACAAGAGTAGAAATTATGC	
Ap-ABF	ATTATTACACCTGCATTATCTTGTAGATAGAGACCTTCGG	
Ap-ABR	ATTTATTTACACCTGCATAACGGCTGAGACCTGACGCTC	
mCh-NcoI-UpF	GCCCCGTAATGCAGAAGAAGAC	
U6HH-DR-R0	TCCTCACGGACTCATCAGTAATTTCAATCACTACTTCGAC	
U6HH-DR-AaR	ATTATTATTACACCTGCATTTGTCCTCACGGACTCATC	
U6HH-DR-F0	CGAAACGAGTAAGCTCGTCTAATTTCTACTAAGTGTAGAT	
U6HH-DR-AaF	ATTATTATTACACCTGCATTTGGACGAAACGAGTAAGC	
U6t-SpeR	AACATTACACTAGTATTGGTTTATCTCATC	
U6DR-AaR	ACATTATTACACCTGCATACATTACAATCACTACTTCGAC	
U6DR-AaF	ATTTATTACACCTGCAACATAATTTCTACTAAGTGTAGAT	
oECA3.1-F	AGATCTAGCTTATCGTGATAGATCTTCCG	
oECA3.1-R	CGGCCGGAAGATCTATCACGATAAGCTAG	
oECA3.2-F	AGATAGCAATTATACTATCAGATATCCGG	
oECA3.2-R	CGGCCCGGATATCTGATAGTATAATTGCT	
oGL2-F	AGATTATGTCAATGGCCGTCGACATGTG	
oGL2-R	CGGCCACATGTCGACGGCCATTGACATA	
oTT4-F	AGATCTATTCACAGGCGACAAGTCGACG	
oTT4-R	CGGCCGTCGACTTGTCGCCTGTGAATAG	
oGL1.1-F	AGATACTGAACAAGAAGAAGACCTCATG	
oGL1.1-R	CGGCCATGAGGTCTTCTTCTTGTTCAGT	
oGL1.2-F	AGATATAGCTAAAAGAGTACCGGGAAGG	
oGL1.2-R	CGGCCCTTCCCGGTACTCTTTTAGCTAT	
oiCBAHS-F	GACCGTTATAGTTACGAGCTTGAGACCATACCTAGGAAGCTTACCATA	

oiCBAHS-R	CTAGTATGGTAAGCTTCCTAGGTATGGTCTCAAGCTCGTAACTATAACGGTCCTAA	
U6-HiF2	AATTTATAAGCTTCGACTTGCCTTCCGCAC	
NH-NcF	ACAACAACCATGGAAATTACTGATGAGTCCGTG	
Sp-BBR22	ACATTATTGAAGACAAGGCCGAGACCGGCTTATTATGC	
DR-BbF	ATTTAACGAAGACGGTGCATAATTTCTACTAAGTGT	
ECA3-1-NGSF	GGAGTGAGTACGGTGTGCATTATTCTGGTTCATTTGTCTAC	NGS
ECA3-1-NGSR	GAGTTGGATGCTGGATGGAATTATTAAAAAGGCTAGGTTC	
ECA3-2-NGSF	GGAGTGAGTACGGTGTGCAGACTAATGCTGAGAAGGC	
ECA3-2-NGSR	GAGTTGGATGCTGGATGGTAGGGGAAAGAGAGAAACC	
GL2-NGSF	GGAGTGAGTACGGTGTGCGGAAGAAGGAGTGCGAGGA	
GL2-NGSR	GAGTTGGATGCTGGATGGGAGAGAGGGCTGGAGAGGA	
TT4-NGSF	GGAGTGAGTACGGTGTGCTCTACTTTCATTTCCTTTGGC	
TT4-NGSR	GAGTTGGATGCTGGATGGTGTCCAGAGAAGGAGCCAT	
ECA3-1-IDF	AAGCATCACTCAGGGACA	RFLP analysis
ECA3-1-IDR	CGTTTAGCCAAAGAAGGT	
ECA3-2-IDF	TCTTTCGTATTGGCTTTG	
ECA3-2-IDR	CAGTCGGAATAATCAGCA	
GL1-1-IDF	GAACCGCATCGTCAGAAA	Sanger sequencing
GL1-1-IDR	GAAATAACCCACCAACAC	
GL1-2-IDF	TCAATGAAACTCAACCGATGT	
GL1-2-IDR	AGTAGTTGCCATTAGGAC	
At5G40330-F	TCTTCTGGTATACTTGCTCTTACTTCTC	Off-target analysis
At5G40330-R	ATGTAGCCAGATTATGTATGTTGTAGAT	
At1G22640-F	GGTACTTATTCATACATCCTCTGTTTCT	
At1G22640-R	CTCGTTATCTGTTCTTCCTGGTAATCTC	
At5G14750-F	CATTTGCTCAAAATTAGTTTCTCTACTC	
At5G14750-R	TCGAGATTTTCGTTTCTTCTGATGTTTC	

Table S15. Sequences of target sites

Table S15. Sequences of target sites

Gene	Target	PAM	Guide
AtECA3	ECA3-1	TTTC	CTAGCTTATCGTGATAGATCTTCC
	ECA3-2	TTTA	AGCAATTATACTATCAGATATCCG
AtGL2	GL2	TTTG	TATGTCAATGGCCGTCGACATGT
AtTT4	TT4	TTTA	CTATTCACAGGCGACAAGTCGAC
AtGL1	GL1-1	TTTC	ACTGAACAAGAAGAAGACCTCAT
	GL1-2	TTTG	ATAGCTAAAAGAGTACCGGGAAG

Table S16. List of Cas12a vectors generated in this study

Table S16. List of Cas12a vectors generated in this study

Cloning vector	Cas12a	crRNA cassette	Target	Final vector
pBG-Lb01	LbCas12a	U6-tGly-crRNA-HDV-U6t	ECA3-1	pLb01-ECA3-1
			ECA3-2	pLb01-ECA3-2
			GL2	pLb01-GL2
			TT4	pLb01-TT4
			GL1-1	pLb01-GL1-1
			GL1-2	pLb01-GL1-2
pBG-ttLb	ttLbCas12a	U6-crRNA-HDV-U6t	ECA3-1	pttLb-ECA3-1
			ECA3-2	pttLb-ECA3-2
			GL2	pttLb-GL2
			TT4	pttLb-TT4
			GL1-1	pttLb-GL1-1
			GL1-2	pttLb-GL1-2
pBG-ttLb01	ttLbCas12a	U6-tGly-crRNA-HDV-U6t	ECA3-1	pttLb01-ECA3-1
			ECA3-2	pttLb01-ECA3-2
			GL2	pttLb01-GL2
			TT4	pttLb01-TT4
			GL1-1	pttLb01-GL1-1
			GL1-2	pttLb01-GL1-2
pBG-ttLb02	ttLbCas12a	U6-HH-crRNA-HDV-U6t	ECA3-1	pttLb02-ECA3-1
			ECA3-2	pttLb02-ECA3-2
			GL2	pttLb02-GL2
			TT4	pttLb02-TT4
			GL1-1	pttLb02-GL1-1
			GL1-2	pttLb02-GL1-2
pBG-ttLbU01	ttLbCas12a Ultra	U6-tGly-crRNA-HDV-U6t	ECA3-1	pttLbU01-ECA3-1
			ECA3-2	pttLbU01-ECA3-2
			GL2	pttLbU01-GL2
			TT4	pttLbU01-TT4
			GL1-1	pttLbU01-GL1-1
			GL1-2	pttLbU01-GL1-2
pBG-AsU01	AsCas12a Ultra	U6-tGly-crRNA-HDV-U6t	ECA3-1	pAsU01- ECA3-1
			ECA3-2	pAsU01- ECA3-2
			GL2	pAsU01-GL2
			TT4	pAsU01-TT4
			GL1-1	pAsU01-GL1-1
			GL1-2	pAsU01-GL1-2
pBG-ttAsU01	ttAsCas12a Ultra	U6-tGly-crRNA-HDV-U6t	ECA3-1	pttAsU01- ECA3-1
			ECA3-2	pttAsU01- ECA3-2
			GL2	pttAsU01-GL2
			TT4	pttAsU01-TT4
			GL1-1	pttAsU01-GL1-1
			GL1-2	pttAsU01-GL1-2

pBG-ttLbU03	ttLbCas12a Ultra	RPS5A-HH-crRNA-HDV-HSPt	GL1-2	pttLbU03-GL1-2
pBG-ttLbU04	ttLbCas12a Ultra	UBQ1-HH- crRNA-HDV-HSPt	GL1-2	pttLbU04-GL1-2
pBG-ttLbU05	ttLbCas12a Ultra	RPS5A-U6-tGly-crRNA-HDV-HSPt	GL1-2	pttLbU05-GL1-2
pBG-ttLbU06	ttLbCas12a Ultra	UBQ1-U6-tGly-crRNA-HDV-HSPt	GL1-2	pttLbU06-GL1-2
pBG-ttLbU	ttLbCas12a Ultra	U6-crRNA-HDV-U6t	ECA3-1	pttLbU-ECA3-1
			GL2	pttLbU-GL2
			TT4	pttLbU-TT4
			GL1-2	pttLbU-GL1-2
pBG-ttLbUV2	ttLbCas12a Ultra V2	U6-crRNA-HDV-U6t	ECA3-1	pttLbU2-ECA3-1
			GL2	pttLbU2-GL2
			TT4	pttLbU2-TT4
			GL1-2	pttLbU2-GL1-2

The red letters represent simplifed names of the cassettes.
